# Muscle growth by sarcomere divisions

**DOI:** 10.1126/sciadv.adw9445

**Published:** 2025-07-09

**Authors:** Clement Rodier, Ian D. Estabrook, Eunice HoYee Chan, Gavin Rice, Vincent Loreau, Stefan Raunser, Dirk Görlich, Benjamin M. Friedrich, Frank Schnorrer

**Affiliations:** ^1^ CNRS, IBDM, Turing Centre for Living Systems, Aix Marseille University, Marseille, France.; ^2^ Cluster of Excellence “Physics of Life,” Technical University Dresden, Dresden, Germany.; ^3^ Max Planck Institute of Molecular Physiology, Dortmund, Germany.; ^4^ Max Planck Institute for Multidisciplinary Sciences, Göttingen, Germany.

## Abstract

The sarcomere is the elementary contractile unit of muscles. Adult muscle cells are large and chain thousands of sarcomeres into long periodic myofibrils that attach to the skeleton. During development, muscle cells must increase in length to maintain the mechanical connection to the growing skeleton. How muscles add new sarcomeres to facilitate muscle growth is unknown. Using live imaging and high-throughput image analysis, we have now tracked the sarcomere components during the developmental growth of *Drosophila* muscle and found that individual sarcomeres divide along the myofibril tension axis into daughter sarcomeres. This way, new sarcomeres can be inserted into contractile and mechanically intact myofibrils. We propose that sarcomere division is triggered by tension and local sarcomere damage originating from skeletal growth and muscle contractions. Sarcomere divisions repair damaged sarcomeres, ensure their mechanical integrity, and synchronize sarcomere addition with skeletal growth during animal development.

## Teaser

When muscle cells enlarge during developmental growth, they divide their contractile sarcomeres into daughter sarcomeres.

## INTRODUCTION

**Fig. 1.**
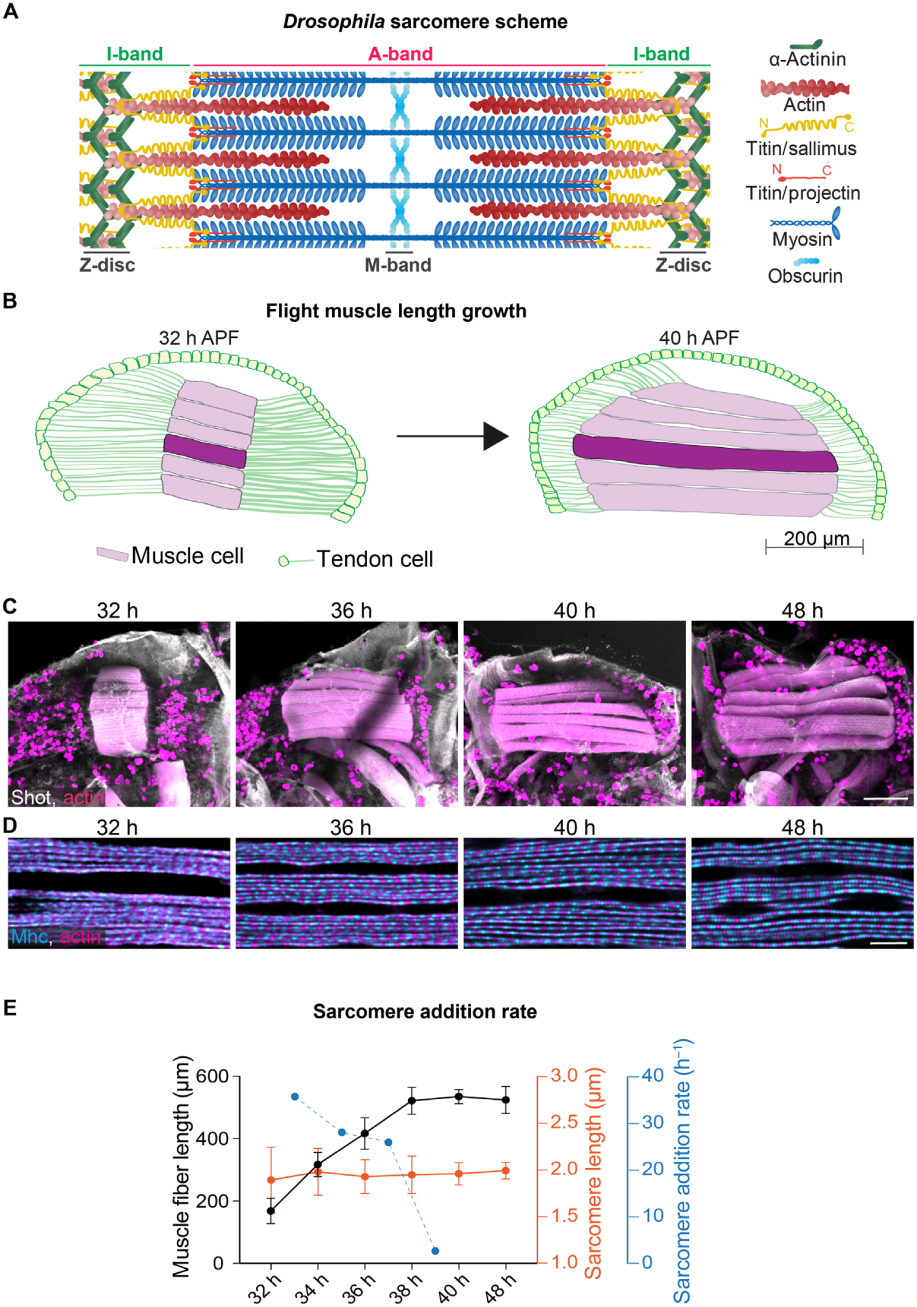
Muscle growth and sarcomere addition. (**A**) Schematic of a mature *Drosophila* flight muscle sarcomere bordered by two Z-discs containing α-actinin (green), which cross-links polar actin filaments (red). Central bipolar myosin filaments (blue) are cross-linked by obscurin at the M-band and form the A-band region of the sarcomere. The myosin-free zone is the I-band region. The titin homolog Sallimus (Sls) links the Z-disc to the myosin filaments, and the second titin homolog Projectin is located at both ends of the myosin filaments. Scheme was adapted from (*2*) including data from (*21*). (**B**) Schematic of a *Drosophila* hemithorax at pupal stages with the six dorsal longitudinal flight muscles (DLMs) in magenta and the tendon cell epithelium in green. DLM4 is highlighted and was used for quantifications in (E). The stable connection of the flight muscles to the tendon cell extensions (green lines) during the growth phase from 32 to 40 hours after puparium formation (APF). h, hours. (**C** and **D**) Confocal images of pupae of the indicated stages displaying the six flight muscles stained for actin (phalloidin in red) and Shot (anti-Shortstop in gray, also labeling the tendon extensions) in (C). High magnifications displaying the myofibrils stained for actin (red) and myosin [anti–Mhc (myosin heavy chain) in blue] in (D). Scale bars, 100 μm in (C) and 5 μm in (D). Note the marked muscle length increases after 32 hours APF. (**E**) DLM4 fiber length plotted in black and sarcomere length in red. Calculated sarcomere addition rate per myofibril per hour in blue. Error bars indicate SD. Number of scored sarcomeres is >500 from three to seven animals per stage, and DLM4 muscle length was measured from 4 to 15 animals per stage (see data S1).

Sarcomeres are the contractile units of muscles and have a stereotypic length of 2 to 3 μm in mammals (*1*). Muscle cells can be centimeters long, with thousands of sarcomeres connected in series to form long periodic chains called myofibrils. These bridge across the entire cell, and their contraction moves the skeleton (*2*). During early mammalian development, the fetal cardiomyocytes and skeletal muscle cells have already assembled chains of sarcomeres to pump the fetal blood and move its skeleton. These fetal muscle cells are generally small, matching the small size of the fetal heart and skeleton. As development proceeds, the distance between skeletal elements and the heart size enlarges, which is accompanied by a matched length increase in the skeletal muscle cells (muscle fibers) or, postnatally, cardiomyocytes (*2*–*4*). Because sarcomere length, which is measured between the two bordering Z-discs (Fig. 1A), is stereotypic and remains constant during this growth, a large number of additional sarcomeres need to be added to each existing myofibril. This addition represents a nontrivial problem, as myofibrils are continuously under large mechanical tension, and thus a myofibril would snap if opened in an uncontrolled way (*5*, *6*).

In 1969, Mackay and Harrop (*7*) inserted metal wires into the muscles of young rats and followed their positions during postnatal growth, which suggested that myofibers add new material all along their length. However, to date, the mechanism of how sarcomeres are added is unknown. The most prominent hypothesis, the “end hypothesis,” proposed that new sarcomeres are added exclusively at the terminal ends of myofibrils, where these are stably attached to tendon cells (*8*, *9*). Evidence for this hypothesis dates back to 1938 from experiments counting sarcomere numbers in growing tadpoles (*10*). One reason that made this hypothesis attractive is that the terminal Z-disc of each myofibril is thicker and, in some cases, has a zigzag shape (*11*, *12*), which may allow the insertion of material without breaking the myofibrillar chain (*13*, *14*). However, to our knowledge, there is no direct observational evidence supporting this model. Visualizing sarcomere addition in growing muscles would be conclusive evidence, but this is hard to obtain in a developing mouse or human being. To this end, we reasoned that the problem is more fundamental and ancient than mammalian evolution, as the basic sarcomeric architecture is highly conserved (*15*), and turned to *Drosophila* for studying the process.

## RESULTS

### Flight muscles add sarcomeres rapidly

*Drosophila* dorsal longitudinal indirect flight muscles (here called flight muscles for short) are an ideal model to study developmental muscle growth, as they are composed of only six large muscle cells in each fly hemithorax. After stably attaching to tendons at about 32 hours after puparium formation (APF), each flight muscle cell assembles all of its 2000 myofibrils at once (*16*, *17*). Then, the muscle cells grow and double their length within a few hours of development (Fig. 1B) (*16*, *18*, *19*). To study the process of sarcomere addition in detail, we first quantified the length of the flight muscle fibers and the respective number of sarcomeres every 2 hours of development (Fig. 1, C to E). Our analysis verified that individual sarcomere length remains constant at about 2 μm, while sarcomere regularity increases (Fig. 1D) as has been described (*20*). This analysis identified a particularly fast sarcomere addition phase between 34 and 38 hours APF, with each myofibril containing about 150 sarcomeres at 34 hours and reaching 250 sarcomeres at 38 hours. Thus, each myofibril adds about 100 sarcomeres in 4 hours or 1 sarcomere every 2 min (Fig. 1E). We concluded that the developing insect flight muscle is a good system to study the mechanism of sarcomere addition.

### Flight muscles are not selectively added at the muscle ends

If the end hypothesis of sarcomere addition would apply in this system, then one would expect to find many highly irregular newly added sarcomeres close to the terminal Z-discs. To test this, we performed high-resolution imaging, visualizing actin, myosin, and titin [called Sallimus (Sls), the I-band titin in *Drosophila* (*21*)] at the terminal and central flight muscle regions at 32, 36, and 40 hours APF (fig. S1, A and B). We found the expected maturation of the sarcomere pattern also at the terminal end when development progressed; however, no obvious differences in sarcomere length or regularity were present compared to central muscle regions (fig. S1, A and B), suggesting that sarcomere addition is at least not limited to the myofiber end only.

**Fig. 2.**
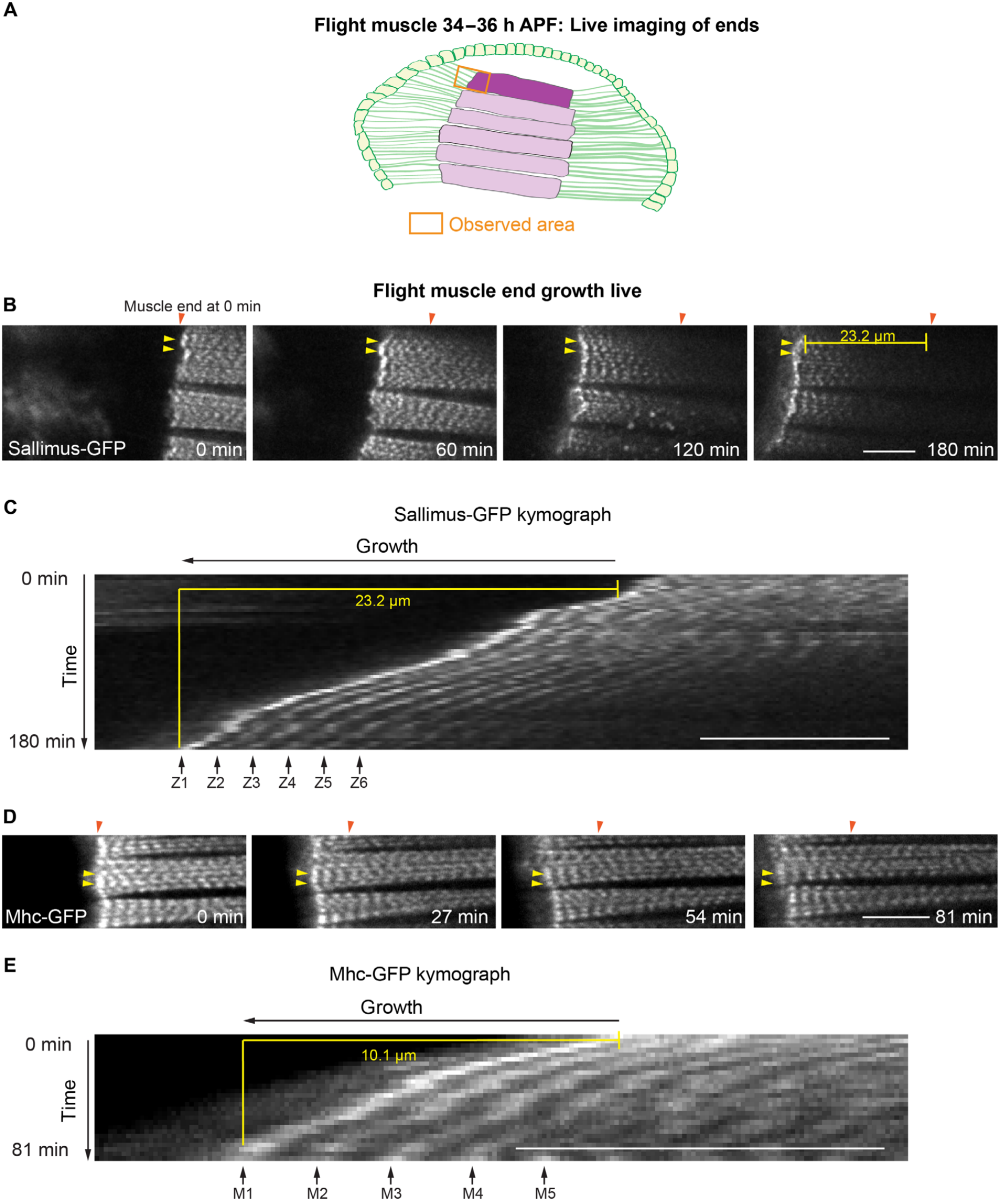
Flight muscle end growth live. (**A**) Schematic of a *Drosophila* hemithorax at 34 to 36 hours APF with the six DLMs in magenta and the tendon cell epithelium in green. DLM1 is highlighted, and the estimated area used for live imaging of myofibril terminal ends is marked by an orange box. (**B** and **C**) About 34 hours APF, a pupa expressing Sls-GFP with a focus on the anterior flight muscle end. Stills from movie S1 are shown (B), and a kymograph of a selected region is shown (C). The myofibril ends, marked with yellow arrowheads, move to the left, while muscle fiber length increases and thus moves away from the reference point marked with the orange arrowhead. No obvious sarcomere addition is seen at the ends. (**D** and **E**) About 36 hours APF, a pupa expressing Mhc-GFP with a focus on the anterior flight muscle end. Stills from movie S2 are shown (D), and a kymograph of a selected region is shown (E). The myofibril ends, marked with yellow arrowheads, move to the left, while muscle fiber length increases and thus moves away from the reference point marked with the orange arrowhead. No obvious sarcomere additions at the ends are seen. Scale bars, 10 μm. At least three pupae were imaged for each genotype. h, hours.

To investigate the dynamics of the terminal sarcomeres directly, we imaged the growing muscle ends in living pupae from about 34 hours APF onward using a titin–green fluorescent protein (GFP) knock-in (Sls-GFP) strain. We found that the terminal Z-discs can be traced over time without any obvious dynamics that may indicate sarcomere addition. Furthermore, no flow of sarcomeres away from the end can be observed in kymographs (Fig. 2, A to C, and movie S1). The latter would be expected if new sarcomeres are only inserted at the terminal ends of the myofibrils. We found the same when imaging flight muscle ends in living pupae from a Myosin heavy chain–GFP knock-in (Mhc-GFP) strain and tracing the terminal myosin filament stacks (Fig. 2, D and E, and movie S2). No obvious sarcomere addition and no flow of myosin stacks were found. Hence, we concluded that flight muscles rapidly add new sarcomeres during their fast growth phase. However, identifying the position of addition prompted a more detailed investigation.

### Building a tool to automatically trace developing sarcomeres

**Fig. 3.**
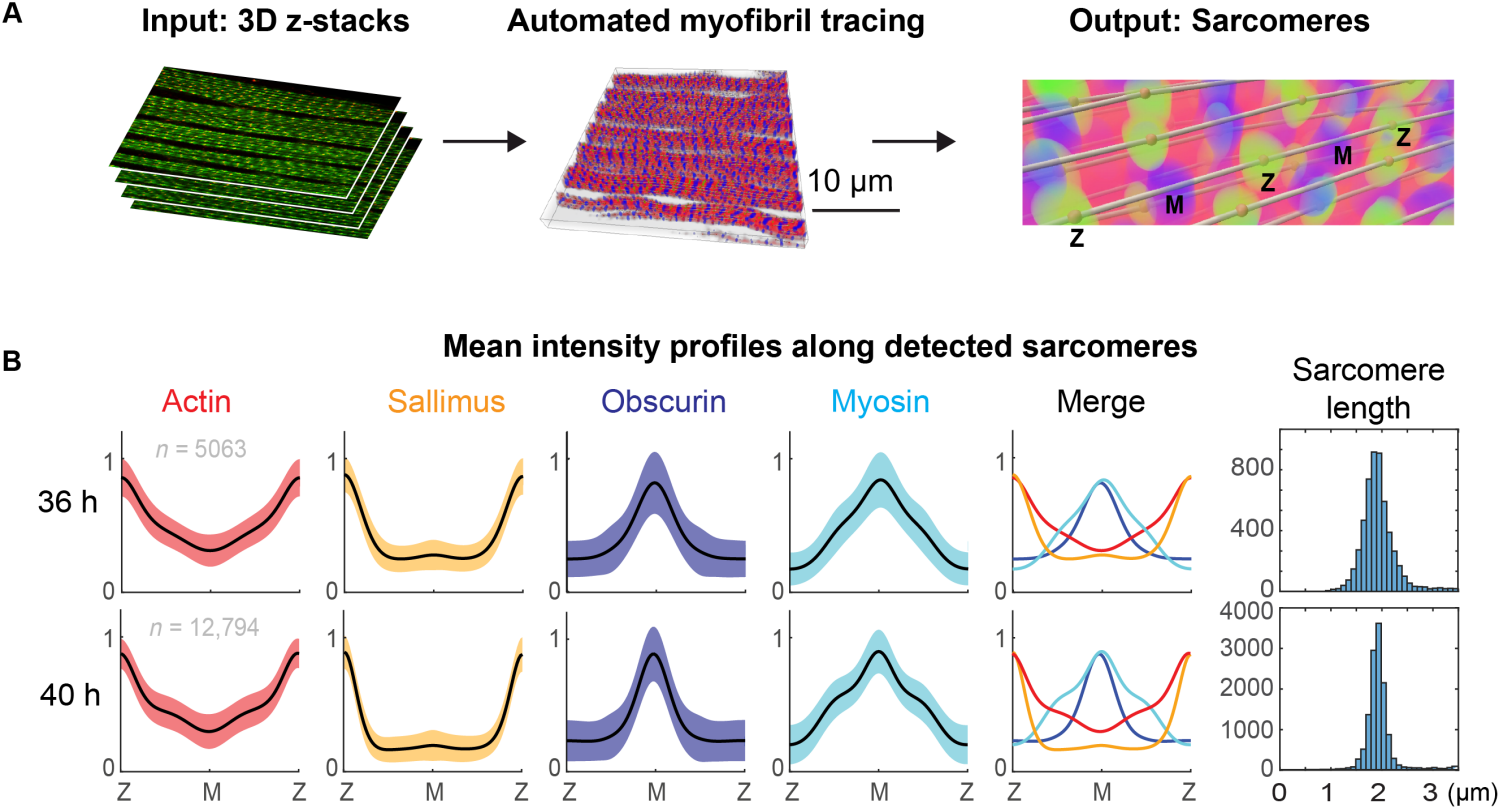
An automated detection tool for flight muscle sarcomeres. (**A**) Summary of automated myofibril tracing from a confocal z-stack of flight muscles stained for sarcomere proteins as input to traced sarcomeres as output; Z-discs and M-bands of a selected myofibril are labeled (see Materials and Methods, fig. S2, and movies S3 and S4). (**B**) Normalized mean intensity profiles computed from detected sarcomeres of actin (phalloidin in red), Sallimus (Sls-Nano2 in orange), Obscurin (anti-Obs in dark blue), and myosin (Mhc-GFP in light blue) shown as mean (black) ± SD (shaded regions) at 36 and 40 hours APF. 36 hours APF: Five samples from two animals, *n* = 5063 sarcomeres; 40 hours APF: Eight samples from three animals, *n* = 12,794 sarcomeres. Histograms of sarcomere length bins are shown to the right. h, hours.

To identify possible sites of sarcomere addition, we developed an automated tool to trace myofibrils in three-dimensional (3D) confocal stacks of flight muscles at pupal stages (Fig. 3A; see Materials and Methods, fig. S2A, and movies S3 and S4). It automatically detects sarcomeres and quantifies sarcomere length and protein distributions for the key sarcomere components actin (labeling the actin filaments), titin N terminus (Sallimus N terminus, labeling the Z-discs), myosin (labeling the myosin filaments), and Obscurin (marking the M-band at the center of the myosin filaments; see Fig. 1A) in an unbiased way at 36 and 40 hours APF (Fig. 3, A to B). We found the expected increase in peak regularity for the sarcomere proteins from 36 to 40 hours APF and the expected 2-μm sarcomere length, when measuring the distance between neighboring Sallimus peaks representing the Z-discs (Fig. 3B). This showed that our 3D detection method accurately identified a large number of sarcomeres (in total, more than 17,000), which would be impractical to trace manually.

### Sarcomeres divide by segregating their myosin filament stacks

**Fig. 4.**
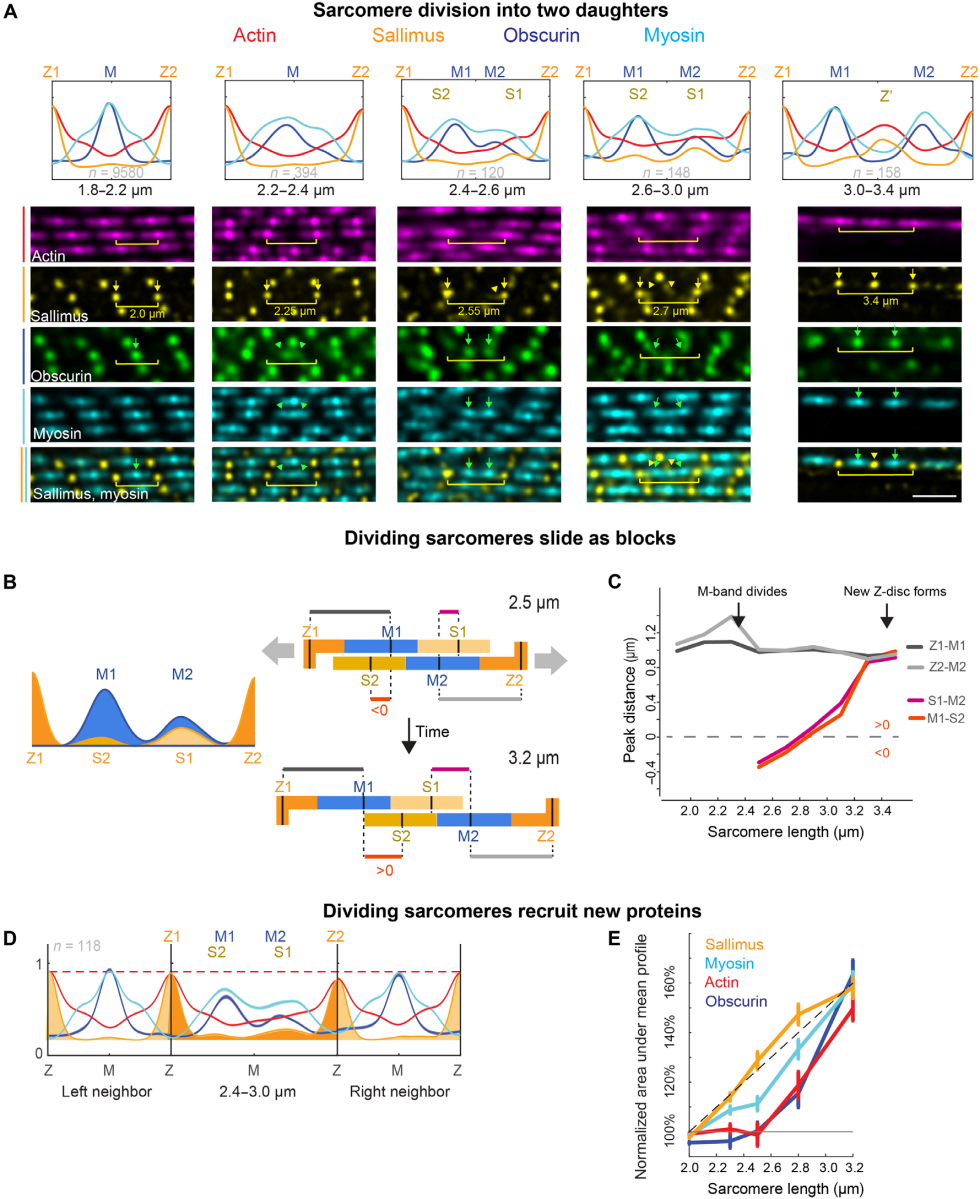
Sarcomere detection reveals sarcomere division by myosin segregation. (**A**) Top: Merged mean intensity profiles for the same proteins as in Fig. 3B sorted by sarcomere length bins using the 40-h APF flight muscles (same eight samples as in Fig. 3; *n* = 120 to 9580 sarcomeres per bin, mean profiles from oriented individual profiles with the larger of the two obscurin signals oriented computationally to the left). Below are representative confocal images with highlighted sarcomeres matching the length categories. Color code used in the profiles above is indicated on the left. Two distinct myosin and Obscurin signals are visible in sarcomeres longer than 2.4 μm (green arrow and arrowheads); concomitantly, Sls is moving away from the Z-discs (marked by yellow arrows) to form a new Z-disc (yellow arrowheads). Scale bar, 2 μm. (**B** and **C**) Peak-to-peak distances of Z1, Z2, S1, S2 (Sallimus), and M1, M2 (Obscurin) peaks computed from the mean intensity profiles shown in (A). Distances Z1-M1 and Z2-M2 (in gray) do not change when sarcomere length increases, while the distances M1-S2 and M2-S1 increase with slope ≈1 (C), suggesting that daughter sarcomeres slide relative to each other as rigid blocks (B; see also movie S5). (**D** and **E**) Intensity profile quantifications for actin, Obscurin, Sallimus, and myosin from all 40-hour sarcomeres that have a length between 2.4 and 3.0 μm and contain right and left neighbors detected by the algorithm (D; *n* = 112). The neighbor sarcomeres are regular, with a slight reduction in actin and Sls intensities at the shared Z-discs. Quantification measures the total protein amounts in the dividing sarcomeres only, normalized to its neighbors. This shows a level increase for all four proteins with increasing sarcomere length (E).

Having validated the detection method, we next searched for deviations from the regular sarcomere pattern, with the motivation that such deviations may indicate sarcomere addition events. We scored sarcomeres with a length between 1.8 and 2.2 μm between neighboring Z-discs as “regular” sarcomeres. In addition, we found many longer sarcomeres (8.6% of 10,479 sarcomeres longer than 1.8 μm), which display unusual yet consistent protein distribution profiles (Fig. 4A and fig. S3): Sarcomeres with a length between 2.2 and 2.4 μm show broader myosin and Obscurin peaks, while sarcomeres ranging from 2.4 to 2.6 μm in length display two distinct Obscurin and two distinct myosin peaks (the Obscurin peaks are labeled M1 and M2 in Fig. 4A). Seeing these data, we hypothesized that the fixed snapshots of increasing sarcomere length are reflecting a temporal event sequence. This would suggest that the widening myosin filament stack (called the A-band; see Fig. 1A) is segregating into two daughter myosin stacks along the tension axis, one to the anterior and one to the posterior side of the muscle fiber. Consistent with the tension-induced division hypothesis, we found that some Sallimus becomes detectable away from the Z-discs, as visible in the stainings and even more prominently in the intensity profiles of the averaged long sarcomeres (new Sls intensities labeled as S1 and S2 in Fig. 4A and fig. S4). This suggests that some Sls were pulled out of the Z-discs toward the center, while the sarcomere starts dividing.

When sarcomere length increases further (>2.6 μm), the myosin and Obscurin stacks move further apart, and the central Sallimus populations assemble into a new Z-disc (called Z’) in the center between the two daughter sarcomeres (Fig. 4A). As expected, we found that the long sarcomeres are homogeneously distributed along the long axis of the flight muscle (the tension axis) (fig. S2B). Together, these snapshots are consistent with the hypothesis that a flight muscle sarcomere can divide into two daughter sarcomeres by segregating its myosin filament stacks along the myofibril tension axis (*17*).

If sarcomere divisions are induced by high tension created by growth of the pupal thorax, then we wondered how the division is molecularly controlled to prevent a fatal rupture of the sarcomere. Sarcomeres are mechanically held together by titin springs, linking the Z-disc to the myosin filaments. *Drosophila* contains two titin homologs, Sallimus and Projectin, with Sallimus linking the Z-disc to the end of the myosin filaments, whereas Projectin is located at the ends of the myosin filaments in flight muscles (see Fig. 1A) (*21*). Thus, segregating the myosin filaments into two distinct blocks should either break the Sallimus/myosin linkage or the Sallimus/Z-disc linkage. Following the pseudotemporal sequence of our detected intensity profiles and quantifying protein amounts in a given sarcomere length bin (Fig. 4B and fig. S4) argue for a combination of both. Thus, localized sarcomere damage triggers the sarcomere divisions. In most cases, the mother sarcomere divides asymmetrically with a larger myosin block (labeled M1) moving to one side (here, computationally placed on the left side in the averages), together with a pronounced Sallimus signal originating from the right Z-disc (S1), while the remaining Sallimus stays at the Z-disc (Z2). At the same time, a smaller myosin block (M2), moving right, segregates together with a smaller Sallimus signal (S2) from the left Z-disc (Z1) (Fig. 4, B and C, and fig. S4). As sarcomere length continues to increase, the mutual distances between Z1, M1, and S1 remain constant, suggesting that these move as an intact block. The same is the case for a second block consisting of Z2, M2, and S2. The distance between M1 and S2 (and thus between the two blocks) continuously increases, exactly as sarcomere length increases (Fig. 4, C and D, and movie S5). In addition, we found that Projectin segregates together with the myosin filaments (fig. S5). From these quantitative data, we concluded that defined local damage of protein interactions allows the two daughter sarcomeres to segregate as two blocks, likely sliding past each other, until the two daughter Z-discs S1 and S2 are brought into contact and subsequently merge to build a new Z-disc separating the two daughter sarcomeres.

To further support the idea that the average sarcomere snapshots represent a temporal division sequence, we quantified total protein amounts in the dividing sarcomere compared to its intact neighbors (Fig. 4D). One would expect that additional sarcomere proteins are recruited over time to the available binding sites to refill the protein levels of both future daughter sarcomeres. In agreement with this prediction, we found that the intensity of the sarcomere proteins increases with increasing sarcomere size (Fig. 4, D and E). The Sls and actin proteins present at the new Z-disc in the middle of 3.2-μm-long sarcomeres reach almost the same levels as at the old Z-disc (Fig. 4E and fig. S4). This recruitment mechanism is compatible with the observed relatively fast turnover rates of myosin, with 50% exchange in about 30 min in sarcomeres of 36-hour APF pupae (fig. S6 and movie S6). Together, this demonstrates that novel sarcomere proteins are recruited to the daughter sarcomeres to refill the myosin stacks, the Obscurin-containing M-bands, and the new Z-disc shared by the daughter sarcomeres.

### Sarcomere division (live)

**Fig. 5.**
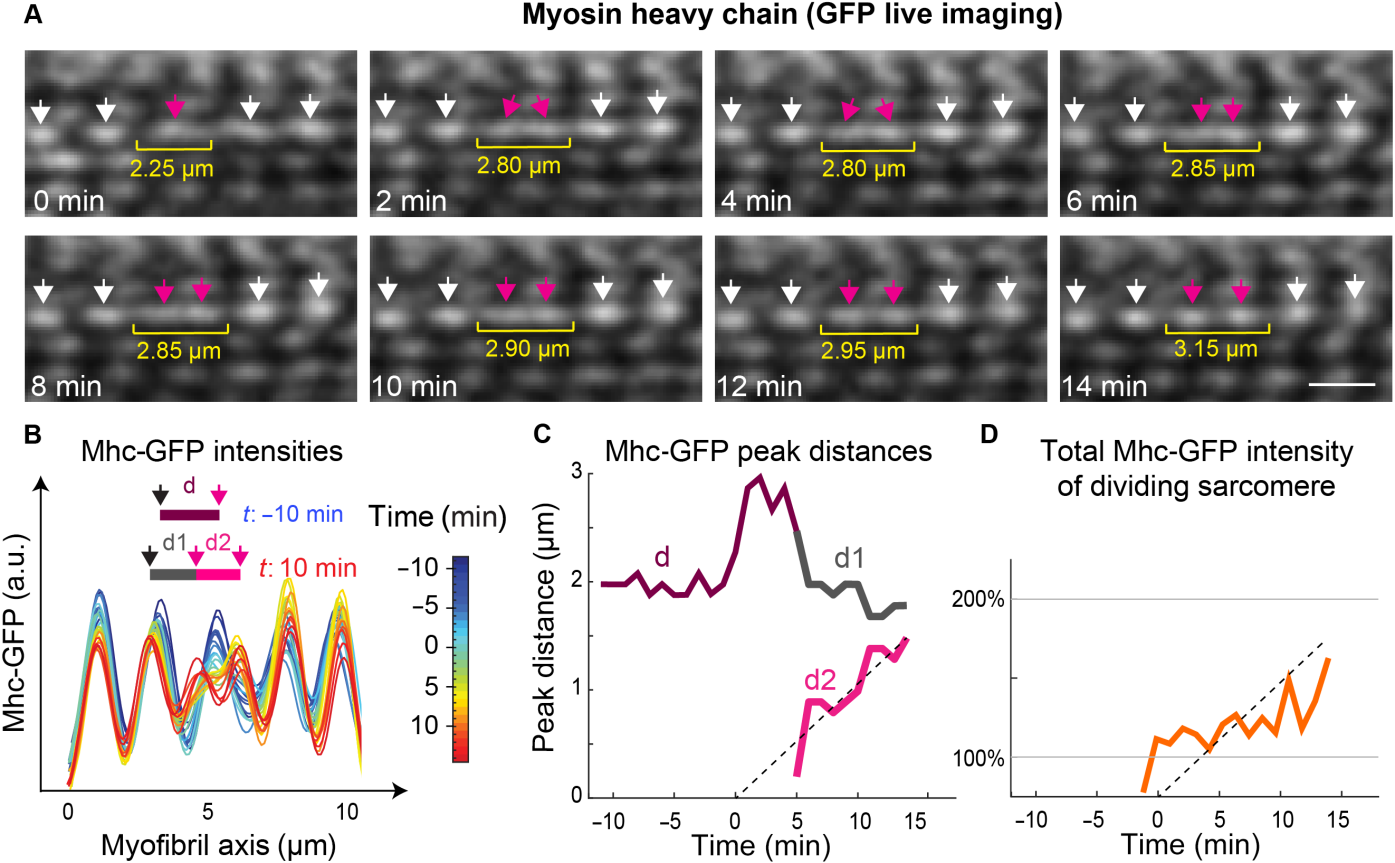
Live imaging of sarcomere division. (**A**) Stills from a time-lapse movie of Mhc-GFP–expressing flight muscles of a 36-hour APF pupa acquired with two-photon microscopy (see movie S7). Arrows indicate the myosin filament stacks of one selected myofibril. Sarcomere length is indicated with the yellow brackets. The dividing myosin filament stack is labeled with magenta arrows. The increase in sarcomere length correlates with the segregation of the two new myosin peaks. Scale bar, 2 μm. (**B**) Mhc-GFP intensity profiles along the highlighted myofibril in (A) for different color-coded time points. Distances between Mhc-GFP peaks are highlighted for two frames (*t*: −10 min in blue and *t*: 10 min in red). Time 0 min indicates the putative start of the division event. a.u., arbitrary units; d, distance. (**C**) Interpeak distances of intensity profiles from (B) as a function of time. Color code corresponds to the inset in (B). (**D**) Normalized total Mhc-GFP intensity of the tracked dividing sarcomere. Note the increase in MhcGFP amount over time.

Thus far, we collected evidence for sarcomere divisions based on fixed images. To directly investigate the temporal dynamics of myosin filament stack segregation during sarcomere division in growing flight muscles, we performed in vivo live imaging using two-photon microscopy of an endogenously tagged functional Mhc-GFP line (Fig. 5A) (*17*, *22*). In these movies, we identified several events, in which a single myosin stack representing one A-band segregated into two separate stacks of lower intensities over the course of 10 to 20 min (Fig. 5, A and B; fig. S7, A to C; and movies S7 and S8). We quantified the distances between the adjacent myosin peaks along the myofibril to track the dividing myosin stack in comparison to its neighbors (Fig. 5, B and C). As soon as the myosin stack divided, its two daughters were pulled away from each other at a speed of ~0.1 μm/min until their distance had increased to about 1.8 μm, which is close to the mature sarcomere length (Fig. 5C and fig. S7). In the course of the division, the intensities of both daughter myosin peaks and the total amount of Mhc-GFP in the daughter sarcomeres increased, again showing that new myosin proteins are recruited to replenish protein levels in the daughter sarcomeres (Fig. 5, B and D, and fig. S7). These data strongly support the sarcomere division mechanism in flight muscles that we identified on the basis of the fixed images (Fig. 4).

### Sarcomere division in larval muscles

Flight muscle sarcomeres are not cross-striated; their myofibrils are isolated (*23*), and hence, sarcomeres in neighboring myofibrils can divide independently. Furthermore, flight muscle myofibrils are not yet fully contractile during the pupal stages that we analyzed above. To investigate whether a similar sarcomere division mechanism also applies during the developmental growth of actively contractile cross-striated muscle types, we chose to investigate the growth of the cross-striated *Drosophila* larval body muscles that resemble mammalian skeletal muscles more closely. At the end of embryogenesis, larval muscle sarcomeres are fully contractile and power the larval movements. During the three larval instars, the larvae strongly increase in length; the segment-spanning ventral longitudinal muscles increase from about 10 sarcomeres in small L1 larvae to about 60 sarcomeres 4 days later in fully grown L3 larvae (*24*, *25*), suggesting an average addition rate of one new sarcomere every 2 hours over 4 days. In contrast to immobile pupae, larvae move vigorously while eating food to fuel larval growth (*24*).

**Fig. 6.**
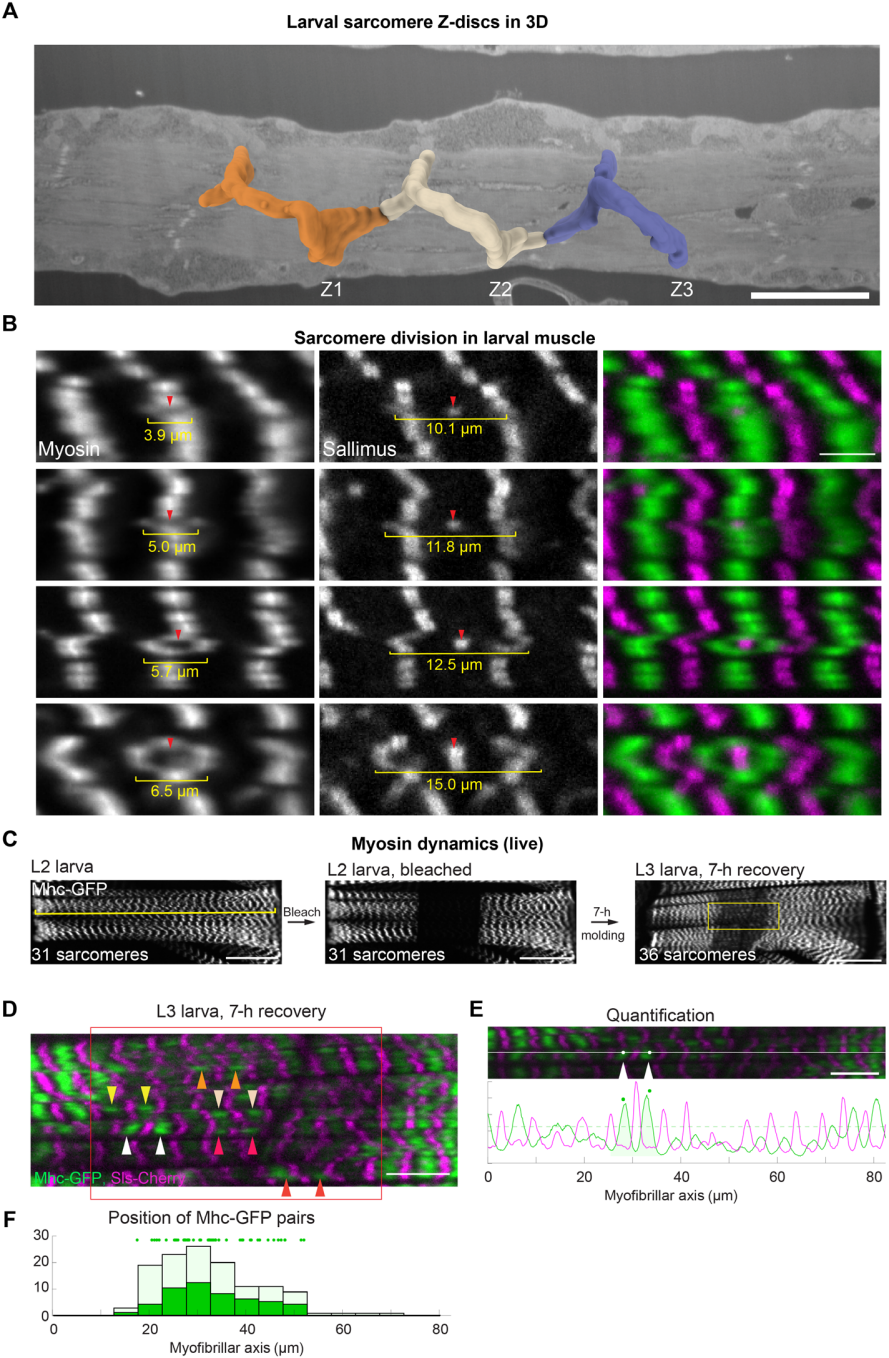
Dividing sarcomeres in larval muscles. (**A**) SEM segmentation of L3 larval Z-discs in a 3D volume starting from the muscle surface using the slices of fig. S9. Each color indicates voxels that were associated with one complete Z-disc at the lateral myofibril edge. See also movie S9. Scale bar, 7 μm. (**B**) Living *Drosophila* L3 larval muscles labeled with Mhc-GFP (green) and Sls-Cherry (magenta) display myosin rings sorted by increasing diameters (yellow), which show increasing amounts of Sls in their center (red arrowheads). The different images were collected from different animals. Scale bar, 5 μm. (**C**) Mhc-GFP in living late L2 larval muscle (dorsal oblique 2), before fluorescence recovery after photobleaching (FRAP; left), after FRAP (middle; FRAP region marked by red rectangle), and after a 7-hour recovery with feeding, resulting in molding to L3 larva (right; approximate region shown as high magnification in (D) is marked with yellow rectangle). The yellow line marks 31 sarcomeres before FRAP, which increases to 36 sarcomeres after a 7-hour recovery (which are more contracted compared to the L2 state). Scale bars, 50 μm. (**D**) High magnification of yellow region in (C) with Mhc-GFP in green and Sls-Cherry in magenta. Mhc-GFP recovers in pairs marked by arrowheads (see also movie S12). Scale bar, 10 μm. (**E**) Automatic quantification of recovered myosin peaks in the bleached region using parallel line scans along the myofibrillar direction (top; white line shows example), and corresponding intensity profiles of Mhc (green) and Sls (magenta) with identified pairs of recovered myosin peaks (bottom; green dots). Scale bar, 10 μm. (**F**) Automatic quantification of the position of the Mhc-GFP peaks (light green) and peaks belonging to a pair (dark green) along the parallel line scans. Note that new Mhc-GFP is recruited throughout the bleached volume. h, hours.

To document the larval sarcomere morphology in detail, we first applied plasma-focused ion beam scanning electron microscopy (pFIB/SEM) and imaged large volumes of larval muscle, resulting in complete 3D reconstructions. The SEM volumes revealed thin myofibrils that were stacked laterally, as expected (fig. S8A). We also found that Z-discs and myosin filament stacks are frequently out of register laterally, with the Z-discs showing frequent Y-shaped branches (fig. S8, A and B). The high contrast of the Z-discs enabled us to generate detailed 3D reconstructions (fig. S9, A to C). We labeled the Z-discs at the edge of the myofibril stacks and segmented their densities toward the center. This allowed us to trace the Z-discs, revealing an extensive, interconnected Z-disc network (Fig. 6A and movie S9). These results inspired us to hypothesize that tensile forces may induce a progression of the Y-shaped branches, similar to the opening of a zipper, which would be consistent with a tension-induced sarcomere division mechanism.

To investigate such a possible mechanism in larval muscle in more detail, we stained larvae expressing Mhc-GFP with phalloidin to label the actin filaments and an N-terminal anti-Sls nanobody to label the Z-discs and imaged them with confocal microscopy, followed by a deconvolution algorithm for optimal *z* resolution. In general, these markers reproduced the cross-striated larval sarcomere morphology built by thin aligned myofibrils. Consistent with our EM data, we frequently identified Z-discs and the myosin filaments as branched Y-shaped structures, sometimes even as double Y-shaped filament stacks that appear as “rings” in one plane (fig. S10A and movie S10).

To rule out that these structures could be a fixation artifact, we next analyzed living intact anesthetized larvae expressing Mhc-GFP or Obscurin-GFP to label the M-band, together with Sallimus-Cherry marking the I-band close to the Z-disc. We found the same myosin stack ring structures as in the fixed images. Analogous to the non–cross-striated flight muscles, we hypothesized that rings with a small diameter might be Y-shaped zippers that have only recently opened, while large rings might be developmentally older. Accordingly, we ordered the rings according to their diameter and assayed how much Sallimus is present in the ring center. If the diameter of the ring is small, then only a little Sallimus is present in its center, while with increasing ring diameter, the amount of central Sls increases to reach the Sls concentration of a regular sarcomere (Fig. 6B and fig. S10, B to D). At the same time, the total amount of Obscurin-GFP present in the segregated myosin stacks doubles, thus replenishing the segregated myosin filament stacks to normal Obscurin levels (fig. S10, B to D). These static images suggest a related sarcomere division mechanism as found in the flight muscles: M-bands, or, possibly, also Z-discs, segregate into two blocks, which eventually mature into two daughter sarcomeres separated by a new Z-disc or M-band in the center of the new sarcomere. The Y-shaped morphology of the junction suggests that the “zipper-like” mechanism can proceed along the short axis of the muscles (transverse to the tension axis), and hence, numerous new sarcomeres can be added in the thin stacked myofibrils throughout the large muscle fiber.

We next attempted to visualize the zipper dynamics in larval muscles directly. We imaged anesthetized living larvae expressing Obscurin-GFP and tracked the M-bands over time. However, within 1 hour, no obvious zipper mechanism was detectable under these conditions (fig. S11A and movie S11). This lack of sarcomere addition is likely caused by reduced sarcomere contractions and the absence of muscle fiber growth during anesthesia. This result is consistent with a key role of larval cuticle growth, to which the muscles are connected, and hence tension exerted on myofibrils, to break protein bonds in the sarcomere and advance sarcomere addition.

To allow for normal muscle contractions and larval growth, we next anesthetized the larvae only transiently and then placed them back into food. To still be able to track sarcomere proteins, we photobleached parts of one muscle to distinguish old from newly synthesized or newly recruited Mhc-GFP (fig. S11B). When photobleaching Mhc-GFP throughout an entire *z* volume of the muscle, the bleached muscle area could be distinguished from neighboring regions for up to 4 days, while the larvae were feeding. Notably, when imaging the bleached area again, we found sites of high Mhc-GFP fluorescence often close to Y-shaped Mhc branches or as localized pairs, suggesting that newly synthesized Mhc-GFP was incorporated locally in neighboring myosin stacks of two daughter sarcomeres that segregated recently and replenished their myosin content (fig. S11B). This is consistent with the hypothesis that the sarcomere division can propagate at the Y-shaped stacks using a zipper mechanism.

However, we realized that the Mhc-GFP recovery under these conditions took days and the larvae grew only a little, likely caused by damage using high laser intensity during fluorescence recovery after photobleaching (FRAP) or toxicity caused by the repetitive ether anesthesia. Thus, we repeated the experiment using milder anesthesia and milder bleaching in late L2 stage larvae, which we placed back into the food and imaged them only once after. Some of these larvae molted to the L3 stage within a few hours. Under these conditions, we found the addition of new sarcomeres, e.g., five new sarcomeres in 7 hours, matching the above-estimated wild-type growth rate (Fig. 6D). Zooming into the recovered area revealed several pairs of high Mhc-GFP signal through the recovering volume, which occur with high statistical significance (*P* < 10^−4^; Fig. 6E and movie S12). We quantified the position of these Mhc-GFP pairs with respect to the bleached muscle area and found that these occur homogeneously in the bleached area (Fig. 6F). This is consistent with the zipper mechanism progressing at multiple regions in the striated larval muscles. Together, these data provide strong evidence that cross-striated larval sarcomeres divide by segregating myosin filament stacks into daughter stacks anywhere throughout the growing muscle fiber.

## DISCUSSION

The identified sarcomere division mechanism here allows the addition of new sarcomeres into existing myofibrils anywhere along the growing muscle fiber. This is a fundamentally different mechanism compared to the end hypothesis, which proposed that new sarcomeres are exclusively inserted at the terminal Z-discs of muscle fiber ends (*8*, *9*, *13*, *14*). The latter may still apply under certain circumstances but would require the transport of all newly synthesized sarcomere components to the muscle fiber ends and the damage to be limited to the ends.

**Fig. 7.**
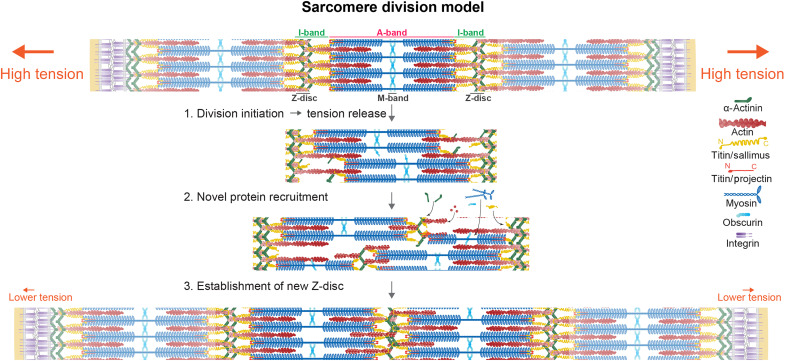
Sarcomere division model. Working model of the tension-induced sarcomere division in *Drosophila* flight muscle sarcomeres. The growth of the skeleton, marked by integrin-based attachments, induces high tension, which triggers the division of the highlighted middle sarcomere. Thus, the sarcomere number increases from three to four. For details, see Discussion.

How are sarcomere additions regulated? Every sarcomere division requires the controlled breakage of protein-protein interaction bonds, i.e., controlled sarcomere damage, to allow segregation of the sarcomere components, without an uncontrolled break of the entire myofibril. We propose that controlled damage is triggered by the high mechanical tension, which is built up by skeletal growth that stretches the attached muscles, while they keep contracting. As a consequence, tensile forces in the sarcomere increase homogeneously along the myofibrils, following Newton’s third law, until a threshold is passed and a sarcomere division is triggered or the zipper progresses forward. Which protein may sense this high tension to initiate the division? The best candidate is the giant I-band titin Sallimus, which, in analogy to the mammalian titin, is responsible for most of the passive sarcomere tension and is stretched across the I-band region, when a sarcomere is stretched (*5*, *25*–*27*). We propose a model in which high mechanical forces cause the breakage of some of the Sallimus—Z-disc linkages—or, also, some Sallimus—myosin linkages—on both Z-discs flanking the dividing sarcomere. This allows the segregation of some bipolar myosin filaments to the right and some to the left, thus adaptively reducing tension within the myofibril (Fig. 7; step 1: division initiation). This division initiation creates free binding sites that allow the recruitment of new sarcomere proteins, making the division process irreversible (Fig. 7; step 2: novel protein recruitment). During the entire process, myosin can still interact with actin filaments, which prevents the uncontrolled rupture of the entire myofibril. Last, the centrally located Sallimus, actin, and Z-disc components establish a new Z-disc between the segregated myosin stacks (Fig. 7; step 3: establishment of a new Z-disc).

All experiments presented in this paper strongly suggest that a rise in tension induced by the skeletal growth triggers the divisions, when, for example, the tension across titin/Sls passes a critical threshold. Direct measurements of local differences in molecular tension across Sls, e.g., using our recently developed Sls tension sensors (*25*), are challenging because these measurements are limited to ensemble measurements and hence cannot distinguish between the Sls pool under high force that is still bound and the Sls pool that lost binding to myosin or Z-disc with the current spatial resolution of the fluorescence lifetime imaging (FLIM)–fluorescence resonance energy transfer (FRET) in vivo imaging (*25*). However, this parallel study provides very strong support of the tension-driven addition mechanism, as changing the length of Sls directly affects the sarcomere addition rate. Larvae expressing longer Sls contain fewer sarcomeres while maintaining the same total size and hence add new sarcomeres slower (*25*). This is consistent with the longer Sls being stretched to a longer length before breaking from its binding partners. In contrast, larvae with shorter Sls contain more sarcomeres and thus must add sarcomeres faster (*25*).

An additional piece of evidence also strongly supports the tension-induced addition rate regulation: Adult fly thorax size is not identical between individuals; in particular, female thoraces are larger than male ones (*28*, *29*). However, flight muscle sarcomeres have a standard size, which is in females and males alike. Thus, the size of the thorax determines how many sarcomeres are present per myofibril and thus likely sets the sarcomere addition rate during development. Furthermore, not all of the six dorsal longitudinal flight muscles (DLMs) have the same length because the thorax has an oval shape (see Fig. 1B). Still, sarcomere length is the same in all six DLMs. Last, if flight muscle sarcomere length is reduced by introducing a hyperactive Mhc or troponin mutation, then thorax size remains the same, so addition must be accelerated (*25*). Together, these observations strongly support our hypothesis that mechanical tension triggers the divisions and hence sets the addition rate.

If tension triggers sarcomere addition, then why do active sarcomere contractions not continuously trigger sarcomere additions in adults? The important difference is that skeletal growth is stretching the muscles, thus elongating the sarcomeres and stretching the I-band titin. However, the myosin-based active force during sarcomere contraction compresses and not stretches titin, as sarcomeres do shorten during contraction. Thus, passive tension caused by skeletal growth but not active myosin force triggers the addition events.

Is a comparable sarcomere division mechanism present in other striated muscles? EM studies of growing crab muscles (*Callinectes sapidus*, an arthropod) showed structures that can be interpreted as dividing A-bands, consistent with our model (*30*). Hence, the proposed sarcomere division mechanism here is likely conserved in arthropods. In mammalian muscles, the mechanism may vary, as sarcomeres should break at their weakest point and mammalian sarcomere architecture displays important differences compared to the insect one (*31*). Mammalian sarcomeres contain in addition to Obscurin the strong M-band cross-linker myomesin, a protein not present in insects (*32*, *33*), and mammalian titin is tightly bound to myosin along the entire thick filament (*31*). If the myomesin cross-links at the M-band and the titin-myosin interactions are more stable than the α-actinin cross-links at the Z-disc, sarcomere division in mammalian muscle is likely initiated by a tension-induced separation of the Z-discs. This Z-disc “streaming” was frequently observed after either eccentric exercise or mechanical stretching of cardiomyocytes in culture; both protocols generated very high tension and thus local damage on sarcomeres (*34*–*37*). Because both tension protocols can result in the addition of sarcomeres within muscle fibers (*35*, *38*, *39*), a tension-induced sarcomere division mechanism may also occur during mammalian muscle growth.

## MATERIALS AND METHODS

### Experimental design

#### Drosophila *strains and genetics*

Fly stocks and crosses were maintained under standard culture conditions (*40*), and pupae were staged at 27°C to match the well-described developmental time points in the literature (*16*). *Mhc-GFP[weeP26]* (*22*), *sls-GFP* (*19*), or *Obscurin-GFP* (transgenic fosmid fTRG1046) (*41*) were either used homozygously or crossed to *sls[23-Cherry]* (*25*).

#### Flight muscle histology

Pupal flight muscles were dissected and stained as described (*18*, *42*). Briefly, pupae were staged at 27°C, the pupal case was removed with fine forceps, and the pupae were fixed with 4% PFA (paraformaldehyde) in PBST (phosphate-buffered saline with 0.3% Triton-X) for 20 min at room temperature (RT). Pupae were immobilized with insect pins in a silicone dish filled with PBST and dissected. Using fine scissors, the ventral part of each pupa was removed, and the thorax was cut sagittally and isolated from the abdomen, leading to two half thoraces with the flight muscles. These half thoraces were blocked for 30 min at RT (1/30 normal goat serum in PBST) and stained with primary antibodies [anti-Mhc: mouse, 1/100, clone 3e8, Developmental Studies Hybridoma Bank; anti-Shot: guinea pig, 1/500 (*43*)] or fluorescently labeled nanobodies [Sls-Nano2 or Proj-Nano37 (*44*)] by incubating them either at RT for 2 hours or overnight at 4°C. Half thoraces were then washed three times for 10 min in PBST at RT and incubated with secondary antibodies coupled to Alexa Fluor dyes (Alexa Fluor 488, Alexa Fluor 568, and Alexa Fluor 647, Molecular Probes; 1/500) for 2 hours at RT in the dark. To visualize F-actin, rhodamine-phalloidin was added to the secondary antibody solution (Molecular Probes; 1/500 in PBST). Flight muscles and sarcomeres were imaged on a Zeiss LSM 880 confocal microscope with 10×, 20×, 40×, or 63× objectives.

#### Pupal live imaging

Pupae were staged at 27°C and mounted in a custom-made imaging chamber as described (*17*, *45*). Briefly, a small hole in the pupal case above the thorax was cut with sharp forceps (the pupa stays intact). The pupa was placed in the imaging chamber, a thick plastic slide with a groove, turned by about 20°; the abdominal part of the pupae was supported with a small piece of clay to position the thorax straight; and spacers were adjusted such that the thorax just faces the coverslip that contains a drop of 50% glycerol covering the hole to avoid desiccation. Flight muscles and myofibrils were imaged on a Nikon multiphoton microscope (ECLIPSE Ni A1R MP, Nikon) using a 40× high numerical aperture (NA) objective (APO LWD 1.15 WI, Nikon) or a Zeiss NLO-880 multiphoton with a 63× high NA objective.

#### Larva live imaging and FRAP

Larvae were staged at 27°C and anesthetized as described (*46*). Briefly, large L2 or small L3 larvae were exposed to diethyl ether in a hermetic chamber for 90 s (milder anesthesia) or 3 min (stronger anesthesia). Then, larvae were gently placed on a slide using a wet brush, with the trachea facing the bottom and the dorsal oblique muscles facing the coverslip for imaging. Imaging was done using an Olympus spinning disk (Yokogawa W1) coupled with a FRAP module Rapp OptoElectronic (UGA42 firefly) using a 60× objective (UPLSAPO60XS2 NA 1.3 WD 0.3) with Olympus silicon immersion oil.

##### Imaging

Overview images of dorsal oblique muscles in L2 or L3 larvae were acquired with a 488-nm laser (IX-LAS488-100LSS) and a 561-nm laser (IX-LAS561-100LSS).

##### Movies

Overview movies of dorsal oblique muscles in L2 or L3 larvae were acquired with a 488-nm laser (IX-LAS488-100LSS) and a 561-nm laser (IX-LAS561-100LSS). Muscles were imaged until the larvae started to wake up, usually 30 min to 1 hour after anesthesia.

##### FRAP

FRAP was performed with a Rapp OptoElectronic module and software using a 488-nm laser (Oxxius; 488 nm, 200 mW). An overview image was acquired, and a smaller area was bleached within the entire Z-thickness of the muscle selected. Laser power was adjusted to achieve substantial bleaching, depending on larval size. The larvae were placed back into the fly food to allow recovery and feeding, before anesthetizing and imaging the same area of the bleached muscle again.

#### Deconvolution

The fixed flight muscle and larval muscle images were deconvolved to reduce noise. Deconvolution was done with the Huygens software, using the extension dedicated to confocal analysis. Stacks that were deconvolved had a *z*-step distance of 200 nm between each slice and were acquired at high resolution (1024 pixels × 1024 pixels, 0.03321 µm per pixel for fixed flight muscle images, 0.06589 µm per pixel for larval muscle images, slow scanning speed) for each channel.

#### Automated image analysis of flight muscles for myofibril and sarcomere detection

A custom-made image analysis and feature-detection software was implemented in MATLAB (MathWorks Inc.) to automatically analyze multichannel fluorescence microscopy z-stack images of myofibrils of developing flight muscles in *Drosophila* pupae. Figure S2 describes the subsequent steps of the image analysis pipeline used to detect sarcomeres in these 3D images. An extended documentation of the algorithm has been included in a code repository (doi.org/10.5281/zenodo.10108362).

##### Automatic detection of Z-disc locations

The location of Z-discs was first identified in individual z-stack slices of *Drosophila* DLM4 stained for the N terminus of the Z-disc protein Sallimus with Sls-Nano2. For each slice, a binary mask was generated as the logical AND of individual masks generated by applying a global threshold or an adaptive local threshold (using the MATLAB function “adaptthresh”), respectively (step 1). This combined mask was then used to obtain the precise location of each Z-disc as the intensity-weighted center of mass of each connected component of the mask (step 2). Because of the fine *z*-step size of the images, the same Z-disc is often identified multiple times in neighboring slices. To identify the instances of multiple detections of the same Z-disc, we determined the fluorescence intensity of the Sallimus signal along the *z* direction for each putative Z-disc location previously determined in individual slices. A cubic spline fit to this intensity profile allowed us to assign a subvoxel *z* position to each putative Z-disc location as the closest peak position in this interpolated profile. Last, we combined putative Z-disc locations detected in different slices into a single Z-disc if their distance was smaller than 0.4 μm (with the new location of the combined Z-disc given by the mean of the individual detected locations).

##### Myofibril tracing, based on Z-disc locations

Next, we identified which Z-disc locations correspond to neighboring Z-discs on the same myofibril and thus mark the borders of a single sarcomere. Specifically, for each Z-disc location, we determined a list of nearby Z-disc locations within a distance of 3.5 μm and a manually determined angular range for the orientation of their separation vector (step 4). For each of these pairs of Z-disc locations, each marking a candidate sarcomere, we determined the fluorescence intensity from both the actin and the Obscurin channels along the length of the candidate sarcomere. Linear interpolation was used to determine these intensity profiles from the 3D z-stacks. Among all candidate sarcomeres, those were selected for which the sum of the mean intensities from the actin and the Obscurin channels was maximal (steps 5 and 6). Whenever this automatic selection of sarcomere candidates produced an apparent branching of sarcomere chains, this was corrected by selecting from the two branches the sarcomere candidate with the highest mean intensity.

##### Manual curation

Using a custom-made manual curation tool, the user can cycle through all identified sarcomere candidates (with key information such as raw fluorescence images and intensity line scans displayed) and remove erroneous sarcomeres. All sarcomeres with length above 1.8 μm used for quantitative analysis in this study have been screened and confirmed by a human (steps 7 and 8). For the data presented in figs. S3 and S4, stringent curation was applied with the percentage of removed sarcomeres and the number of sarcomeres after curation as follows: 36 hours APF: of 6771 sarcomere candidates, 25% removed and 5063 remained; 40 hours APF: of 16,336 sarcomere candidates, 22% removed and 12,794 remained.

##### Mean intensity profiles

For all confirmed sarcomeres, we determined the intensity profiles for all channels using a linear interpolation of the voxelated z-stack, thus obtaining linear profiles parameterized by a “sarcomere coordinate” 0 ≤ *s* ≤ 1 in which the endpoints of the sarcomere marked by Z-discs correspond to *s* = 0 and *s* = 1, respectively. To account for variations of fluorescence intensity across myofibrils, intensity profiles were normalized by dividing by the maximal value of a smoothed profile (obtained by a moving average with a window size equal to 1/10th of sarcomere length). The normalized intensity profiles corresponding either to all detected sarcomeres or to a subset representing sarcomeres within a given length range were then averaged to obtain the mean profiles as shown in Figs. 3B and 4A, respectively. Per se, detected sarcomeres lack an orientation, as the ordering of their two endpoints is arbitrary. For Fig. 4A, we accounted for a possible asymmetry of intensity profiles by selecting the sarcomere orientation for which the total obscurin intensity in the left half of the sarcomere (0 ≤ *s* ≤ ^1^/_2_) was maximal.

##### Correction for chromatic aberration

We systematically examined multichannel fluorescence images for indications of chromatic aberrations and did not detect any aberration, except for the 405-nm channel in the ultraviolet spectrum (corresponding to nanobody Sls-Nano2-Dylight405 staining for Sallimus). For the chosen size of the field of view, the chromatic aberration can be assumed to amount to a uniform translation of the 405-nm channel. To precisely determine this shift, we exploited the known colocalization of Sls and peaks in actin intensity. Specifically, we first identified peaks in actin intensity analogous to the detection of Sls dots described above in each slice and then generated a false image by placing Gaussian profiles at each identified actin peak position. We computed the 2D cross-correlation of this generated image with the Sls staining and determined the translation vector for the Sls stain from the maximum of this cross-correlation. Last, the Sls-stained image was shifted by this vector so that the actin and Sls stains colocalized as expected. Typically, the aberration values were at most 5 pixels (0.165 μm) in each of the Cartesian directions.

#### Image analysis of live pupal movies

Time-lapse movies of Mhc-GFP–expressing flight muscles of 36-hour APF pupae were acquired with two-photon microscopy (movies S7 and S8). For Fig. 5 and movie S7, the Mhc-GFP peaks were automatically detected as intensity peaks in small regions of interest of Gaussian-blurred frames. Line scans with a transverse width of 0.6 μm (6 pixels) were computed along the chain of line segments joining these peak positions.

Movies were motion stabilized, Gaussian blurred, and averaged over two neighboring slices. For fig. S7, the selected myofibrils were tracked manually, and Mhc-GFP peak positions were determined from a straight-line scan along the myofibril axis with a transverse width of 0.5 μm (6 pixels). Intensity profiles from line scans were smoothed using the MATLAB “smooth” function.

To determine the recovery time of Mhc-GFP after photobleaching in pupae in fig. S6, we determined the mean fluorescence intensity outside and inside the bleached region, respectively, and computed their ratio, *I*(*t*), as a function of time. We then fitted an exponential recovery to the first 70 min of these time series, using the fit function *I*(*t*) ≈ *I*_max_ {1 – exp [−ln (2) *t/t*_50%_]} with the recovery fraction, *I*_max_, and the recovery half-time, *t*_50%_, as free-fit parameters using the MATLAB function “fminsearch” with initial guess *I*_max_ = 0.55 and *t*_50%_ = 30 min.

#### Image analysis of larval sarcomeres

Regions of interest containing single M-band rings, i.e., bulging openings of M-bands, were identified manually in single slices of two-channel z-stacks of larval muscles expressing Obscurin-GFP and Sallimus-mCherry. Positions of landmarks were then manually specified on these rings using a custom-made annotation tool written in MATLAB. Landmarks used comprise (i) the two junctions at which rings connect to the M-bands above and below and (ii) the two outermost positions of the ring defining its diameter. Ring diameter was defined as the length of the projection of the line segment connecting the two outermost positions on the ring on a line perpendicular to the connection between the Y-crossings (see fig. S10C). Integrated Sallimus and Obscurin signals were obtained as total from line scans with a length of 2 μm along the line passing through the two lateral points of the ring, centered at these lateral points and their midpoint, respectively. Each intensity signal was normalized by a reference intensity computed as average over four line scans of the same length across manually selected Sallimus or Obscurin bands outside of the ring in the same image. Each line scan consists of several parallel scans, which are averaged to reduce noise, corresponding to an effective thickness of line scans equal to 50 and 25% of ring height for the Sallimus and Obscurin signal, respectively, and a manually determined thickness for all reference line scans. The intensity minimum was subtracted from each line scan to account for background intensity.

#### Mhc-GFP pair detection in FRAP experiments

To quantify the Mhc-GFP recovery after FRAP in Fig. 6D, we detected sarcomeres by first identifying Sls peaks in parallel line scans along the myofibril direction, exploiting that Sls recovers faster than Mhc. We then marked those sarcomeres, in which Mhc-GFP signal peaks had both a high maximum intensity and a high total intensity, resulting in the detection of 199 (about 7%) pronounced sarcomere peaks of a total of 2995 sarcomeres. We then screened for pairs of adjacent sarcomeres with pronounced Mhc-GFP peaks that were flanked by sarcomeres without pronounced peaks. The detected number of 23 unique pairs is statistically different from the expected number of pairs for a random distribution of pronounced peaks with the same relative frequency (*P* < 10^−4^). Analysis of three additional larvae automatically detected 14, 21, and 13 unique pairs, respectively, which was again statistically relevant (*P* < 10^−2^ in each case).

#### pFIB/SEM volume imaging

*Drosophila* larvae were placed on ice in PBS, dissected, fileted, and fixed by adding 2% glutaraldehyde and 2.5% PFA in PBS overnight at 4°C. Samples were contrasted for 1 hour in potassium ferrocyanide–reduced osmium (2%), 20 min in thiocarbohydrazide, and then 30 min in 2% osmium before being incubated at 4°C overnight in 1% uranyl acetate. Between each incubation, five 3-min washes in water were done. The samples were incubated in fresh 0.66% lead aspartate at 60°C for 30 min and then dehydrated by a series of 10-min incubations in 20, 50, 70, 90, and 100% ethanol and lastly by glass-distilled acetone twice. Then, the samples were incubated in increasing concentrations of resin for 2 hours each at RT (25, 50, 75, and 100% Durcupan diluted in acetone). The 100% Durcupan was renewed and incubated for 16 hours and then incubated for 48 hours at 60°C for the polymerization of the resin (*47*). The samples were embedded by the flat embedding method between Permanox slides. Stained and embedded samples of *Drosophila* larval filets were mounted on standard 12.5-mm SEM stubs (Plano) with conductive double-sided tape (Plano) and loaded into a Helios Hydra 5 (Thermo Fisher Scientific) for volume imaging. Samples were sputter coated with platinum for 60 s using a custom in-chamber sputter coater (Thermo Fisher Scientific) operated at 1 kV and 60 mA. To identify areas containing myofibrils in the tissue, windows were opened using a xenon plasma operated at 30 kV and 60 nA. Once a site of interest was identified, it was coated with a 1-μm protective layer via the gas injection system. The surface was imaged with the SEM operating in immersion mode at 1.5 kV and 1.6 nA with a through-lens detector set up to measure backscattered electrons with a working distance of 2.5 mm and a pixel size of 4.15 nm. Slicing of the sample surface between SEM scans was performed with an oxygen plasma operating at 30 kV and 610 pA, removing 50 nm of material per slice for a total imaged thickness of 13.6 μm. Automated data collection was carried out using Auto Slice and View 5 (Thermo Fisher Scientific).

#### pFIB/SEM data processing

SEM images were stacked and downscaled 4× in Fiji version 2.14.0 (*48*) to reduce file size. Segmentation of features of interest was done in Dragonfly version 2022.2 (Comet Technologies Canada) via a combination of artificial intelligence–driven and manual segmentation. Visualization of the stack along with segmentations was done in ChimeraX version 1.8 (*49*), and a Gaussian filter was applied to smooth segmentations for visualization.

### Statistical analysis

For Fig. 3B, we pooled data from five different samples from two 36-hour APF pupae resulting in a total of 5063 individual sarcomeres and eight different samples from three 40-hour APF pupae resulting in 12,794 sarcomeres. For Fig. 4A, we used the same 40-hour APF data as for Fig. 3B, with the following total numbers of individual sarcomeres in the different length bins: 1.8 to 2.2 μm: *n* = 9580; 2.2 to 2.4 μm: *n* = 394; 2.4 to 2.6 μm: *n* = 120; 2.6 to 3.0 μm: *n* = 148; and 3.0 to 3.4 μm: *n* = 158. For the projectin Proj-Nano37 data shown in fig. S5, we pooled data from five samples from two 40-hour APF pupae, with the following total numbers of individual sarcomeres in the different length bins: 1.8 to 2.2 μm: *n* = 5298; 2.2 to 2.6 μm: *n* = 35; 2.6 to 3.0 μm: *n* = 55; and 3.0 to 3.4 μm: *n* = 41. Error bars denote either the SD or SEM as indicated.

For fig. S10D, we analyzed 221 Obscurin rings from 13 different larvae. Individual data points of normalized intensities were binned according to ring diameter (small: <4 μm, medium: 4 to 6 μm, and large: >6 μm; with respective counts of 53, 114, and 54). The values for *n*, *P*, and the specific statistical test performed for each experiment are included in the appropriate figure and figure legend and the source data table containing all the measurements used for the shown analysis.

## References

[1] M. E. Llewellyn, R. P. J. Barretto, S. L. Delp, M. J. Schnitzer, Minimally invasive high-speed imaging of sarcomere contractile dynamics in mice and humans. Nature 454, 784–788 (2008). 18600262 10.1038/nature07104 PMC2826360

[2] N. M. Luis, F. Schnorrer, Mechanobiology of muscle and myofibril morphogenesis. Cells Dev. 168, 203760 (2021). 34863916 10.1016/j.cdev.2021.203760

[3] T. R. Heallen, Z. A. Kadow, J. Wang, J. F. Martin, Determinants of cardiac growth and size. Cold Spring Harb. Perspect. Biol. 12, a037150 (2020). 31615785 10.1101/cshperspect.a037150 PMC7050588

[4] G. Kardon, Development of the musculoskeletal system: Meeting the neighbors. Development 138, 2855–2859 (2011). 21693508 10.1242/dev.067181

[5] W. A. Linke, Titin gene and protein functions in passive and active muscle. Annu. Rev. Physiol. 80, 389–411 (2018). 29131758 10.1146/annurev-physiol-021317-121234

[6] Q. Mao, A. Acharya, A. Rodríguez-delaRosa, F. Marchiano, B. Dehapiot, Z. Al Tanoury, J. Rao, M. Díaz-Cuadros, A. Mansur, E. Wagner, C. Chardes, V. Gupta, P.-F. Lenne, B. H. Habermann, O. Theodoly, O. Pourquié, F. Schnorrer, Tension-driven multi-scale self-organisation in human iPSC-derived muscle fibers. eLife 11, e76649 (2022). 35920628 10.7554/eLife.76649 PMC9377800

[7] B. Mackay, T. J. Harrop, An experimental study of the longitudinal growth of skeletal muscle in the rat. Acta Anat. (Basel) 72, 38–49 (2004). 10.1159/000143234 5799961

[8] D. J. Dix, B. R. Eisenberg, Myosin mRNA accumulation and myofibrillogenesis at the myotendinous junction of stretched muscle fibers. J. Cell Biol. 111, 1885–1894 (1990). 2229178 10.1083/jcb.111.5.1885 PMC2116343

[9] P. E. Williams, G. Goldspink, The effect of immobilization on the longitudinal growth of striated muscle fibres. J. Anat. 116, 45–55 (1973). 4798240 PMC1271549

[10] C. C. Speidel, Studies of living muscles. I. Growth, injury and repair of striated muscle, as revealed by prolonged observations of individual fibers in living frog tadpoles. Am. J. Anat. 62, 179–235 (1938).

[11] H. J. Green, A. G. Griffiths, J. Ylänne, N. H. Brown, Novel functions for integrin-associated proteins revealed by analysis of myofibril attachment in *Drosophila*. eLife 7, e35783 (2018). 30028294 10.7554/eLife.35783 PMC6092120

[12] K. M. Vakaloglou, G. Chrysanthis, M. A. Rapsomaniki, Z. Lygerou, C. G. Zervas, IPP complex reinforces adhesion by relaying tension-dependent signals to inhibit integrin turnover. Cell Rep. 14, 2668–2682 (2016). 26972014 10.1016/j.celrep.2016.02.052

[13] M. Balakrishnan, S. F. Yu, S. M. Chin, D. B. Soffar, S. E. Windner, B. L. Goode, M. K. Baylies, Cofilin loss in *Drosophila* muscles contributes to muscle weakness through defective sarcomerogenesis during muscle growth. Cell Rep. 32, 107893 (2020). 32697999 10.1016/j.celrep.2020.107893 PMC7479987

[14] M. Ganassi, S. Badodi, K. Wanders, P. S. Zammit, S. M. Hughes, Myogenin is an essential regulator of adult myofibre growth and muscle stem cell homeostasis. eLife 9, e60445 (2020). 33001028 10.7554/eLife.60445 PMC7599067

[15] S. B. Lemke, F. Schnorrer, Mechanical forces during muscle development. Mech. Dev. 144, 92–101 (2017). 27913119 10.1016/j.mod.2016.11.003

[16] M. L. Spletter, C. Barz, A. Yeroslaviz, X. Zhang, S. B. Lemke, A. Bonnard, E. Brunner, G. Cardone, K. Basler, B. H. Habermann, F. Schnorrer, A transcriptomics resource reveals a transcriptional transition during ordered sarcomere morphogenesis in flight muscle. eLife 7, e34058 (2018). 29846170 10.7554/eLife.34058 PMC6005683

[17] M. Weitkunat, A. Kaya-Çopur, S. W. Grill, F. Schnorrer, Tension and force-resistant attachment are essential for myofibrillogenesis in *Drosophila* flight muscle. Curr. Biol. 24, 705–716 (2014). 24631244 10.1016/j.cub.2014.02.032

[18] A. Kaya-Çopur, F. Marchiano, M. Y. Hein, D. Alpern, J. Russeil, N. M. Luis, M. Mann, B. Deplancke, B. H. Habermann, F. Schnorrer, The Hippo pathway controls myofibril assembly and muscle fiber growth by regulating sarcomeric gene expression. eLife 10, e63726 (2021). 33404503 10.7554/eLife.63726 PMC7815313

[19] Z. Orfanos, K. Leonard, C. Elliott, A. Katzemich, B. Bullard, J. Sparrow, Sallimus and the dynamics of sarcomere assembly in *Drosophila* flight muscles. J. Mol. Biol. 427, 2151–2158 (2015). 25868382 10.1016/j.jmb.2015.04.003

[20] O. Loison, M. Weitkunat, A. Kaya-Çopur, C. Nascimento Alves, T. Matzat, M. L. Spletter, S. Luschnig, S. Brasselet, P.-F. Lenne, F. Schnorrer, Polarization-resolved microscopy reveals a muscle myosin motor-independent mechanism of molecular actin ordering during sarcomere maturation. PLOS Biol. 16, e2004718 (2018). 29702642 10.1371/journal.pbio.2004718 PMC5955565

[21] F. Schueder, P. Mangeol, E. H. Chan, R. Rees, J. Schünemann, R. Jungmann, D. Görlich, F. Schnorrer, Nanobodies combined with DNA-PAINT super-resolution reveal a staggered titin nanoarchitecture in flight muscles. eLife 12, e79344 (2023). 36645127 10.7554/eLife.79344 PMC9886278

[22] Z. Orfanos, J. C. Sparrow, Myosin isoform switching during assembly of the *Drosophila* flight muscle thick filament lattice. J. Cell Sci. 126, 139–148 (2013). 23178940 10.1242/jcs.110361

[23] C. Schönbauer, J. Distler, N. Jährling, M. Radolf, H.-U. Dodt, M. Frasch, F. Schnorrer, Spalt mediates an evolutionarily conserved switch to fibrillar muscle fate in insects. Nature 479, 406–409 (2011). 22094701 10.1038/nature10559

[24] F. Demontis, N. Perrimon, Integration of insulin receptor/Foxo signaling and dMyc activity during muscle growth regulates body size in *Drosophila*. Development 136, 983–993 (2009). 19211682 10.1242/dev.027466 PMC2727562

[25] V. Loreau, W. H. Koolhaas, E. H. Chan, P. De Bossier, N. Brouilly, S. Avosani, A. Sane, C. Pitaval, S. Reiter, N. M. Luis, P. Mangeol, A. C. Von Philipsborn, J.-F. Rupprecht, D. Görlich, B. H. Habermann, F. Schnorrer, Titin-dependent biomechanical feedback tailors sarcomeres to specialised muscle functions in insects. Sci. Adv. 11, eads8716 (2025). 40344069 10.1126/sciadv.ads8716 PMC12063666

[26] Y. Li, A. L. Hessel, A. Unger, D. Ing, J. Recker, F. Koser, J. K. Freundt, W. A. Linke, Graded titin cleavage progressively reduces tension and uncovers the source of A-band stability in contracting muscle. eLife 9, e64107 (2020). 33357376 10.7554/eLife.64107 PMC7781594

[27] J. A. Rivas-Pardo, Y. Li, Z. Mártonfalvi, R. Tapia-Rojo, A. Unger, Á. Fernández-Trasancos, E. Herrero-Galán, D. Velázquez-Carreras, J. M. Fernández, W. A. Linke, J. Alegre-Cebollada, A HaloTag-TEV genetic cassette for mechanical phenotyping of proteins from tissues. Nat. Commun. 11, 2060 (2020). 32345978 10.1038/s41467-020-15465-9 PMC7189229

[28] R. B. Huey, B. Moreteau, J.-C. Moreteau, P. Gibert, G. W. Gilchrist, A. R. Ives, T. Garland, J. R. David, Sexual size dimorphism in a *Drosophila* clade, the *D. obscura* group. Fortschr. Zool. 109, 318–330 (2006). 10.1016/j.zool.2006.04.003 16978850

[29] E. J. Rideout, M. S. Narsaiya, S. S. Grewal, The sex determination gene transformer regulates male-female differences in *Drosophila* body size. PLOS Genet. 11, e1005683 (2015). 26710087 10.1371/journal.pgen.1005683 PMC4692505

[30] S. S. Jahromi, M. P. Charlton, Transverse sarcomere splitting. A possible means of longitudinal growth in crab muscles. J. Cell Biol. 80, 736–742 (1979). 457766 10.1083/jcb.80.3.736 PMC2110374

[31] D. Tamborrini, Z. Wang, T. Wagner, S. Tacke, M. Stabrin, M. Grange, A. L. Kho, M. Rees, P. Bennett, M. Gautel, S. Raunser, Structure of the native myosin filament in the relaxed cardiac sarcomere. Nature 623, 863–871 (2023). 37914933 10.1038/s41586-023-06690-5 PMC10665186

[32] M. Gautel, The sarcomeric cytoskeleton: Who picks up the strain? Curr. Opin. Cell Biol. 23, 39–46 (2011). 21190822 10.1016/j.ceb.2010.12.001

[33] P. Young, E. Ehler, M. Gautel, Obscurin, a giant sarcomeric Rho guanine nucleotide exchange factor protein involved in sarcomere assembly. J. Cell Biol. 154, 123–136 (2001). 11448995 10.1083/jcb.200102110 PMC2196875

[34] L. Carlsson, J.-G. Yu, M. Moza, O. Carpén, L.-E. Thornell, Myotilin—A prominent marker of myofibrillar remodelling. Neuromuscul. Disord. 17, 61–68 (2007). 17056257 10.1016/j.nmd.2006.09.007

[35] J.-G. Yu, B. Russell, Cardiomyocyte remodeling and sarcomere addition after uniaxial static strain in vitro. J. Histochem. Cytochem. 53, 839–844 (2005). 15995142 10.1369/jhc.4A6608.2005

[36] J.-G. Yu, L.-E. Thornell, Desmin and actin alterations in human muscles affected by delayed onset muscle soreness: A high resolution immunocytochemical study. Histochem. Cell Biol. 118, 171–179 (2002). 12189520 10.1007/s00418-002-0427-x

[37] J.-G. Yu, L. Carlsson, L.-E. Thornell, Evidence for myofibril remodeling as opposed to myofibril damage in human muscles with DOMS: An ultrastructural and immunoelectron microscopic study. Histochem. Cell Biol. 121, 219–227 (2004). 14991331 10.1007/s00418-004-0625-9

[38] R. Lynn, D. L. Morgan, Decline running produces more sarcomeres in rat vastus intermedius muscle fibers than does incline running. J. Appl. Physiol. 77, 1439–1444 (1994). 7836150 10.1152/jappl.1994.77.3.1439

[39] D. L. Morgan, J. A. Talbot, The addition of sarcomeres in series is the main protective mechanism following eccentric exercise. J. Mech. Med. Biol. 02, 421–431 (2002).

[40] J. Avellaneda, C. Rodier, F. Daian, N. Brouilly, T. Rival, N. M. Luis, F. Schnorrer, Myofibril and mitochondria morphogenesis are coordinated by a mechanical feedback mechanism in muscle. Nat. Commun. 12, 2091 (2021). 33828099 10.1038/s41467-021-22058-7 PMC8027795

[41] M. Sarov, C. Barz, H. Jambor, M. Y. Hein, C. Schmied, D. Suchold, B. Stender, S. Janosch, V. V. Kj, R. Krishnan, A. Krishnamoorthy, I. R. Ferreira, R. K. Ejsmont, K. Finkl, S. Hasse, P. Kämpfer, N. Plewka, E. Vinis, S. Schloissnig, E. Knust, V. Hartenstein, M. Mann, M. Ramaswami, K. VijayRaghavan, P. Tomancak, F. Schnorrer, A genome-wide resource for the analysis of protein localisation in *Drosophila*. eLife 5, e12068 (2016). 26896675 10.7554/eLife.12068 PMC4805545

[42] M. Weitkunat, F. Schnorrer, A guide to study *Drosophila* muscle biology. Methods 68, 2–14 (2014). 24625467 10.1016/j.ymeth.2014.02.037

[43] D. Strumpf, T. Volk, Kakapo, a novel cytoskeletal-associated protein is essential for the restricted localization of the neuregulin-like factor, vein, at the muscle-tendon junction site. J. Cell Biol. 143, 1259–1270 (1998). 9832554 10.1083/jcb.143.5.1259 PMC2133081

[44] V. Loreau, R. Rees, E. H. Chan, W. Taxer, K. Gregor, B. Mußil, C. Pitaval, N. M. Luis, P. Mangeol, F. Schnorrer, D. Görlich, A nanobody toolbox to investigate localisation and dynamics of *Drosophila* titins and other key sarcomeric proteins. eLife 12, e79343 (2023). 36645120 10.7554/eLife.79343 PMC9886281

[45] S. B. Lemke, F. Schnorrer, In vivo imaging of muscle-tendon morphogenesis in *Drosophila* pupae. J. Vis. Exp. 57312, (2018). 10.3791/57312 PMC5912364 29443094

[46] P. Kakanj, S. A. Eming, L. Partridge, M. Leptin, Long-term in vivo imaging of *Drosophila* larvae. Nat. Protoc. 15, 1158–1187 (2020). 32042177 10.1038/s41596-019-0282-z

[47] T. J. Deerinck, E. A. Bushong, M. H. Ellisman, A. Thor, “Preparation of biological tissues for serial block face scanning electron microscopy (SBEM),” in *National Center for Microscopy and Imaging Research* (University of California, 2022).

[48] J. Schindelin, I. Arganda-Carreras, E. Frise, V. Kaynig, M. Longair, T. Pietzsch, S. Preibisch, C. Rueden, S. Saalfeld, B. Schmid, J.-Y. Tinevez, D. J. White, V. Hartenstein, K. Eliceiri, P. Tomancak, A. Cardona, Fiji: An open-source platform for biological-image analysis. Nat. Methods 9, 676–682 (2012). 22743772 10.1038/nmeth.2019 PMC3855844

[49] E. C. Meng, T. D. Goddard, E. F. Pettersen, G. S. Couch, Z. J. Pearson, J. H. Morris, T. E. Ferrin, UCSF ChimeraX: Tools for structure building and analysis. Protein Sci. 32, e4792 (2023). 37774136 10.1002/pro.4792 PMC10588335

