# Supplementary material for: Muscle growth by sarcomere divisions
Source: Sci Adv. 2025 Jul 9;11(28):eadw9445. doi: 10.1126/sciadv.adw9445 (PMC12239948; doi:10.1126/sciadv.adw9445)
Supplement: Supplementary file 1 — Figs. S1 to S11 Legends for movies S1 to S12 Legends for data S1 to S5 [file sciadv.adw9445_sm.pdf]

Science Advances
AAAS

Supplementary Materials for

Muscle growth by sarcomere divisions

Clement Rodier *et al.*

Corresponding author: Frank Schnorrer, frank.schnorrer@univ-amu.fr;
Benjamin M. Friedrich, benjamin.m.friedrich@tu-dresden.de

Sci. Adv. **11**, eadw9445 (2025)
DOI: 10.1126/sciadv.adw9445

The PDF file includes:

Figs. S1 to S11
Legends for movies S1 to S12
Legends for data S1 to S5

Other Supplementary Material for this manuscript includes the following:

Movies S1 to S12
Data S1 to S5

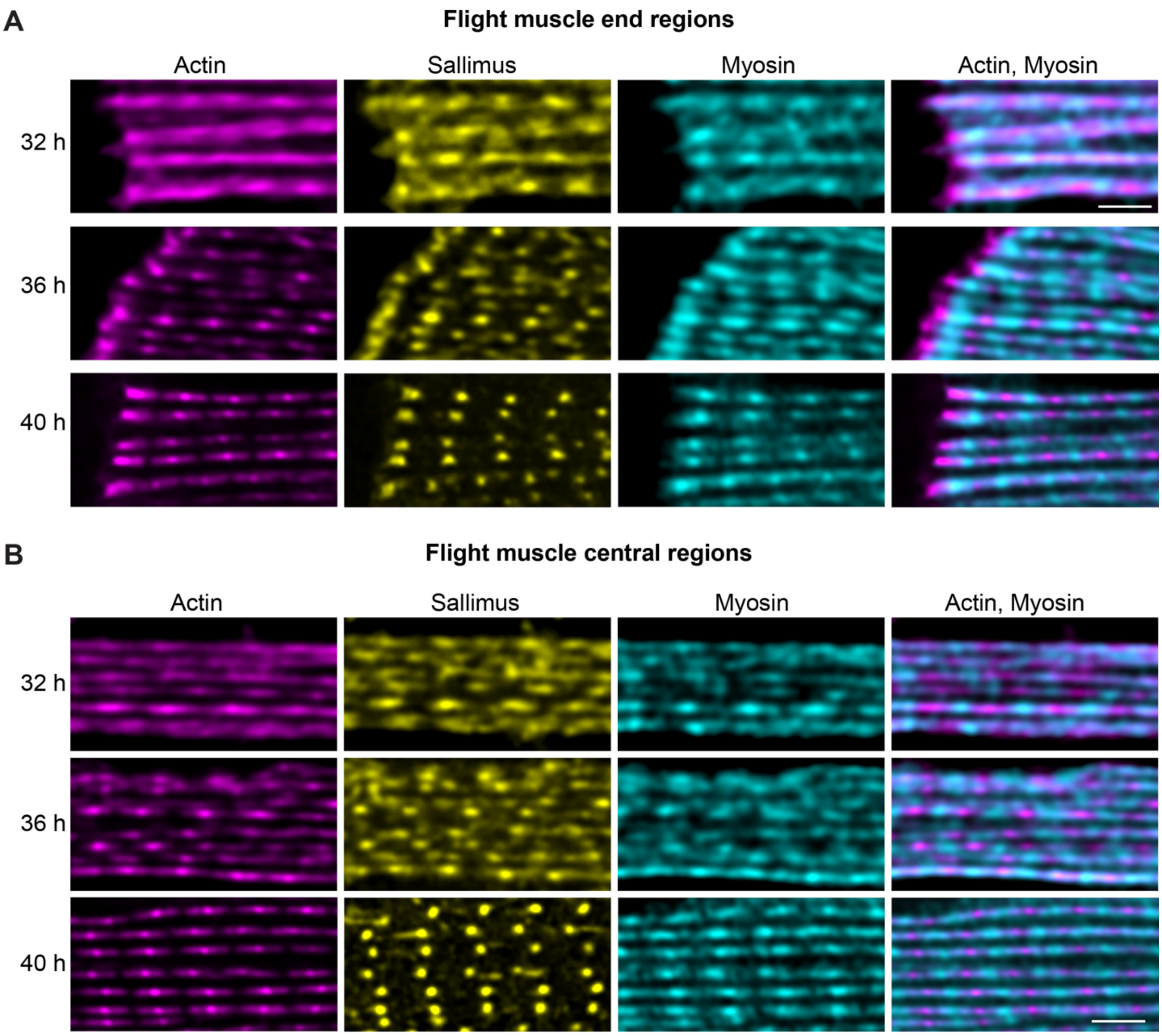

Fig. S1 – Terminal ends vs. center of growing myofibrils.
(A, B). High-resolution confocal images of flight muscle myofibrils at 32 h, 36 h and 40 h APF stained for actin (phalloidin in red), Sallimus (Sls-Nano2 in yellow), myosin (anti-Mhc in cyan) focusing on the myofibrils ends in (A) or central regions in (B). Scale bars are 2 µm. Note the periodic patterns of the sarcomeric components, which become more regular over time. No irregular sarcomeres are present at the terminal ends.

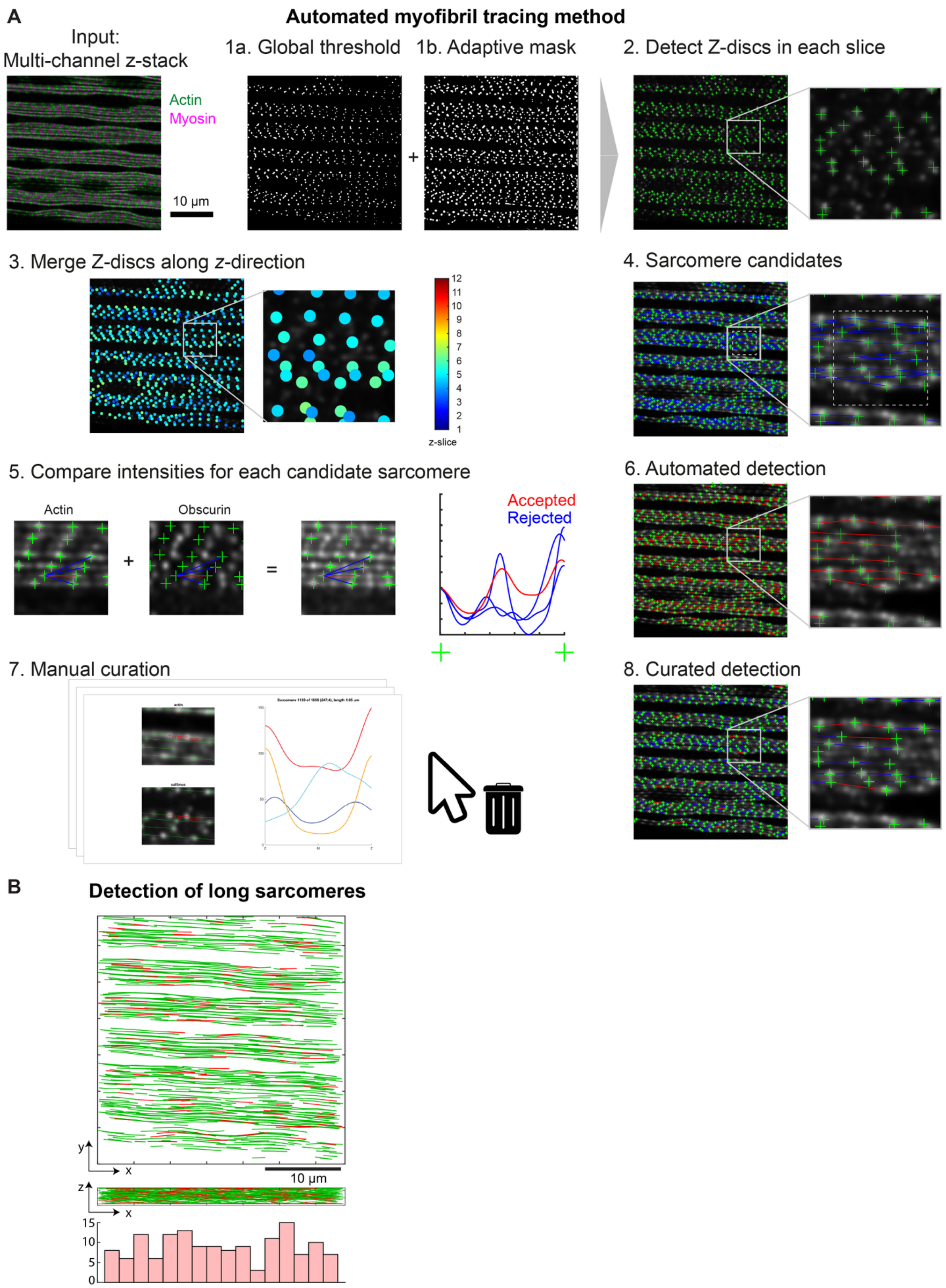

A
Automated myofibril tracing method
Input:
Multi-channel z-stack
Actin
Myosin
10 µm
1a. Global threshold
1b. Adaptive mask
2. Detect Z-discs in each slice
3. Merge Z-discs along z-direction
z-slice
4. Sarcomere candidates
5. Compare intensities for each candidate sarcomere
Actin
Obscurin
Accepted
Rejected
6. Automated detection
7. Manual curation
8. Curated detection
B
Detection of long sarcomeres
10 µm

Fig. S2 – Automated sarcomere detection.

(A) Input: 40 h APF multi-channel confocal microscopy z-stacks of flight muscles stained for Obscurin, actin, myosin and Sallimus, here represented as typical slice with merge of actin (green) and myosin channels (magenta). **Step 1.** Computation of binary masks from the Sallimus channel using global thresholding (1a) and adaptive thresholding (1b). **Step 2.** Z-disc identification in each z-slice as intensity-weighted centers-of-mass of each connected component of a combined mask (green crosses), obtained as logical AND of the masks from step 1. **Step 3.** For each identified Z-disc, its z-position is determined with sub-voxel resolution by fitting a cubic spline to the Sallimus signal along the z-direction. Identified Z-discs from different slices were merged if their Euclidian distance was smaller than 0.4 µm. Shown are detected Z-discs near selected slice with z-position indicated by color code. **Step 4.** Identifying sarcomere candidates (blue lines) by connecting pairs of neighboring Z discs (green crosses) along the myofiber axis within a given tolerance and a maximal length of 3.5 µm. **Step 5.** Automatic selection of true sarcomeres from these candidates, based on the combined fluorescence intensity of actin and Obscurin channels along the sarcomere length. **Step 6.** Result of the automated sarcomere detection. **Step 7.** Manual curation of automated sarcomere detection using a custom-made manual curation tool. **Step 8.** Curated myofibril and sarcomere detection results (red: discarded sarcomeres, blue: retained sarcomeres). **(B)** Spatial distribution of long sarcomeres (red, length > 2.2 µm) and normal sarcomers (green, length < 2.2 µm) present in the entire z-stack are shown (z-depth: 2.4 µm), a single xy plane is shown in panel A (40 h APF). The histogram shows the distribution of long sarcomeres along the image x-axis, which is approximately parallel to the myofibrillar axis. Note the equal distribution of long sarcomeres.

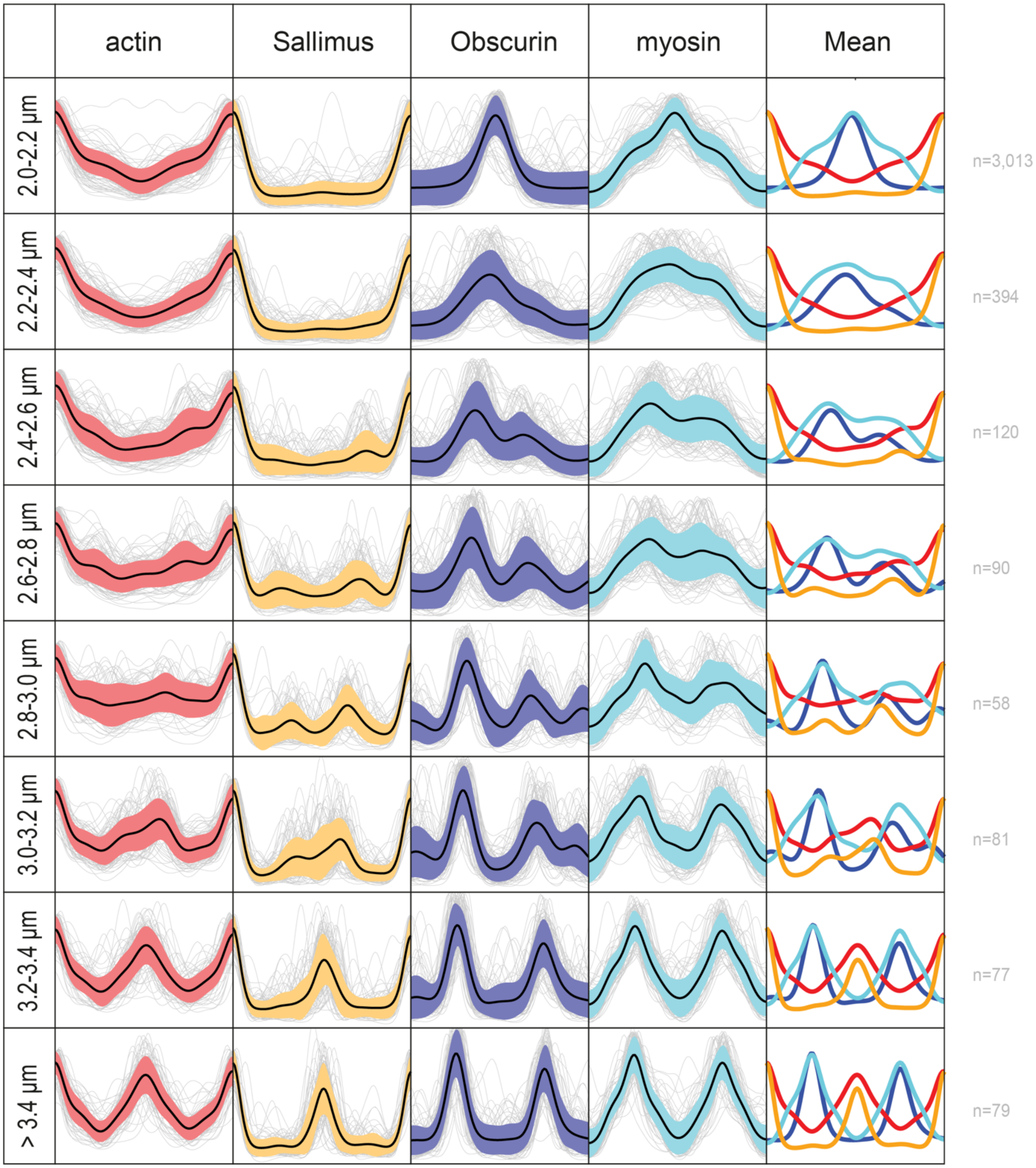

Fig. S3 – Individual and mean intensity profiles of regular and dividing sarcomeres. Grey curves show normalized intensity profiles of individual sarcomeres detected at 40 h APF for actin, Obscurin, Sallimus and myosin, sorted according to sarcomere length (same samples as used for Figs. 3 and 4). Black curves and shaded regions depict mean ± s.d.; the right-most column shows the profiles as mean ± s.e.m.

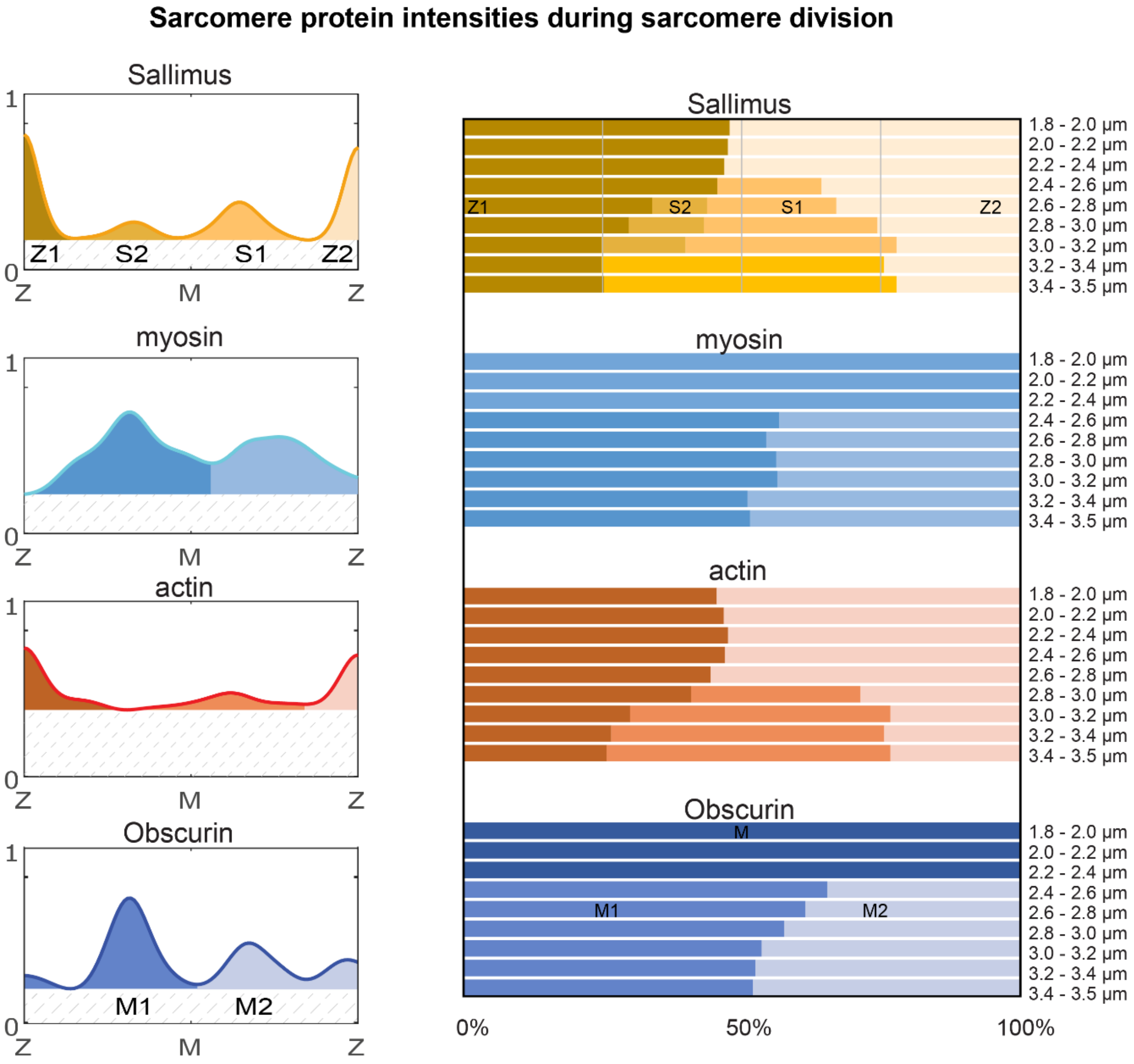

Fig. S4 – Sarcomere protein intensities as peak areas of mean profiles.
Relative areas under the peaks from the mean intensity profiles of sarcomeres within the indicated length bins for Sallimus, myosin, actin, and Obscurin at 40 h APF. Mean profiles were obtained analogously to those shown in Fig. 4A, but using bins as indicated. Different peaks are represented by colors as shown in the example mean profiles to the left. To compute the areas under the peaks, a background intensity given by the minimum of the respective mean profile was subtracted. The relative protein amounts found in each of the peaks for each sarcomere bin size are shown on the right.

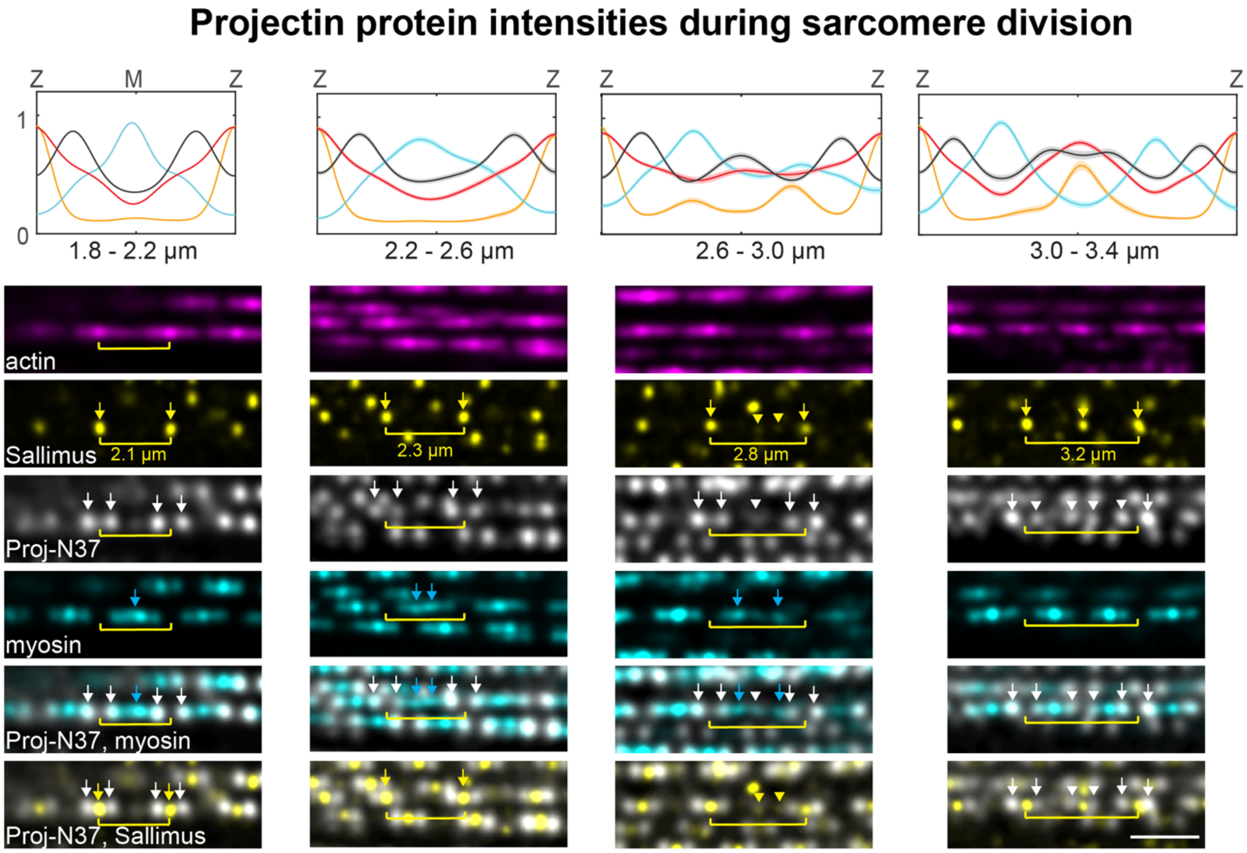

Fig. S5 – Projectin segregates with myosin during sarcomere division.
Top: mean intensity profiles of sarcomere proteins within the indicated sarcomere length bins from flight muscles at 40 h APF stained for actin (phalloidin in red), and C-terminus of Projectin (Proj-Nano37 in white), Sallimus (Sls-Nano2 in yellow) and myosin (Mhc-GFP in cyan); mean ± s.e.m. (shaded regions); 5 samples, 1.8 - 2.2 µm: *n* = 5,298 sarcomeres, 2.2 - 2.6 µm: *n* = 35, 2.6 - 3.0 µm: *n* = 55, 3.0 - 3.4 µm: *n* = 41). Below are representative confocal images with highlighted sarcomeres matching the length categories. Note that the two distinct Projectin peaks (white arrows) labelling the ends of the myosin filaments (blue arrows) in regular sarcomeres (1.8 - 2.2 µm) move with the dividing myosin filaments to form two additional new peaks (white arrowheads) next to the newly emerging Z-disc (yellow arrowhead). Scale bar is 2 µm.

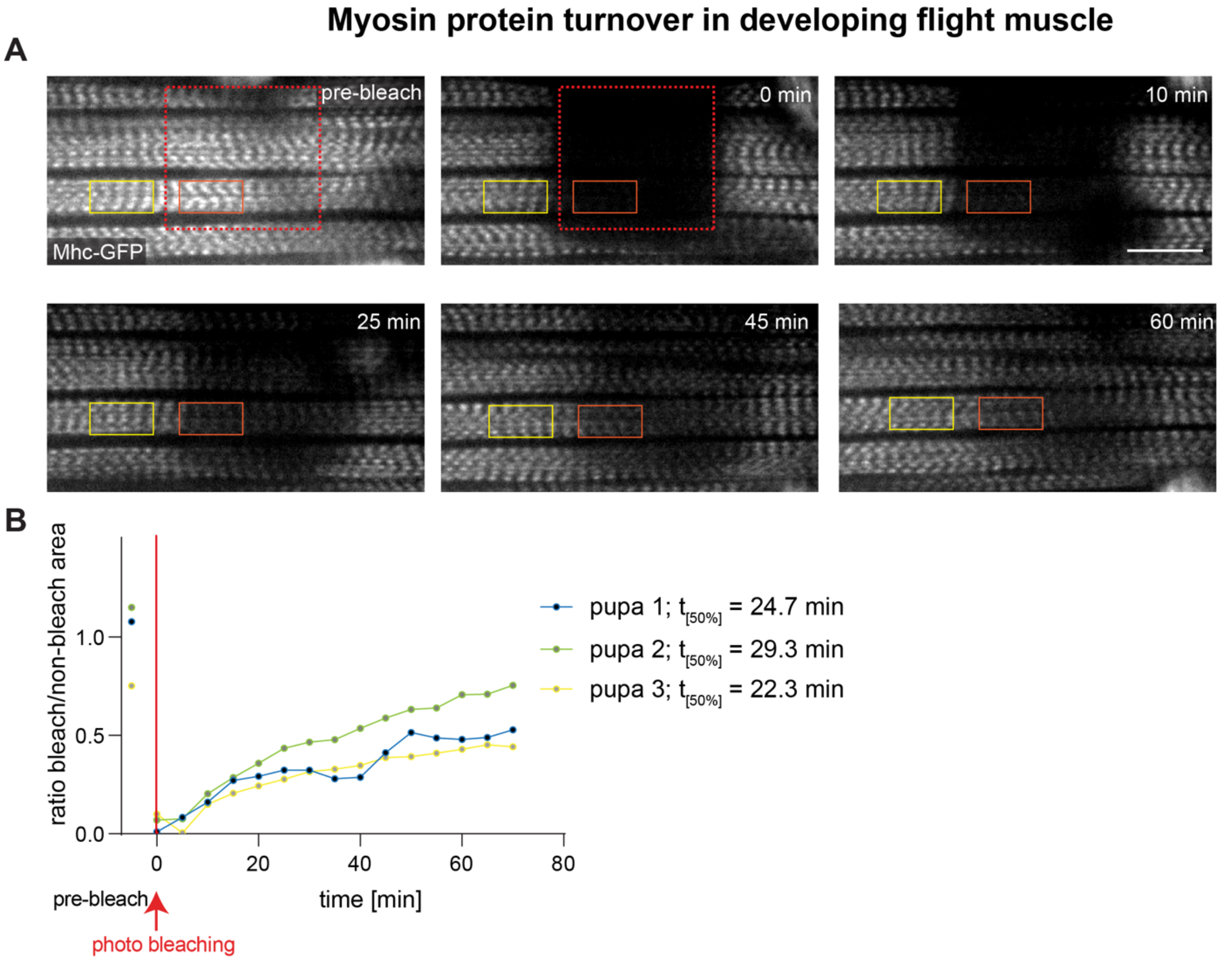

Fig. S6 – Myosin turnover in flight muscles.
(A) Stills from 36 h APF flight muscles expressing Mhc-GFP imaged every 5 min. At 0 min, a square was bleached using the 488 nm laser and recovery of the signal was imaged. Scale bar is 10 µm. (B) Quantification of the Mhc-GFP recovery from 3 representative pupae. $T_{50\%}$ recovery time was calculated (see Methods).

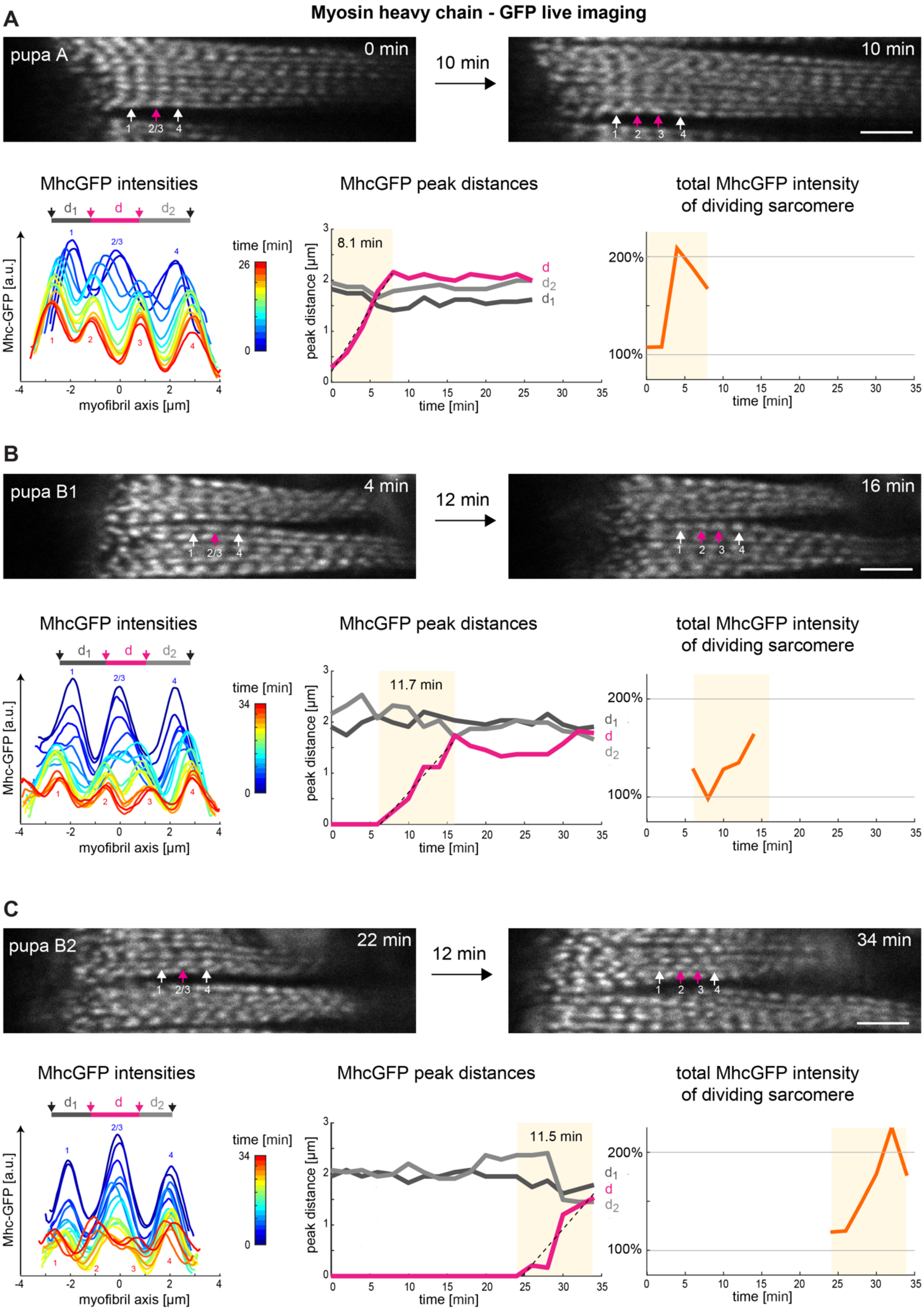
Myosin heavy chain - GFP live imaging
A
pupa A
0 min
10 min
10 min
MhcGFP intensities
MhcGFP peak distances
total MhcGFP intensity of dividing sarcomere
Mhc-GFP [a.u.]
myofibril axis [µm]
time [min]
peak distance [µm]
time [min]
8.1 min
B
pupa B1
4 min
12 min
16 min
11.7 min
C
pupa B2
22 min
12 min
34 min
11.5 min
d
d1
d2
200%
100%

Fig. S7: Additional sarcomere divisions imaged live.
(A-C) Stills from time-lapse movies of Mhc-GFP expressing flight muscles at 36 h APF pupae analogous to Fig. 5 (see Movie S8). In each movie, three sarcomeres labelled 1, 2/3, 4 were manually selected from which the middle one labelled 2/3 will divide into 2 daughters in each movie. Mhc-GFP intensity profiles along the chosen myofibrils are shown below for different color-coded time points (*left*), together with the distances between the highlighted Mhc-GFP peaks (*middle*) and the total Mhc-GFP intensities quantified in the dividing sarcomere. Note that the divisions take about 10 to 15 minutes and the Mhc-GFP intensity increases during the divisions. Scale bars are 5 μm.

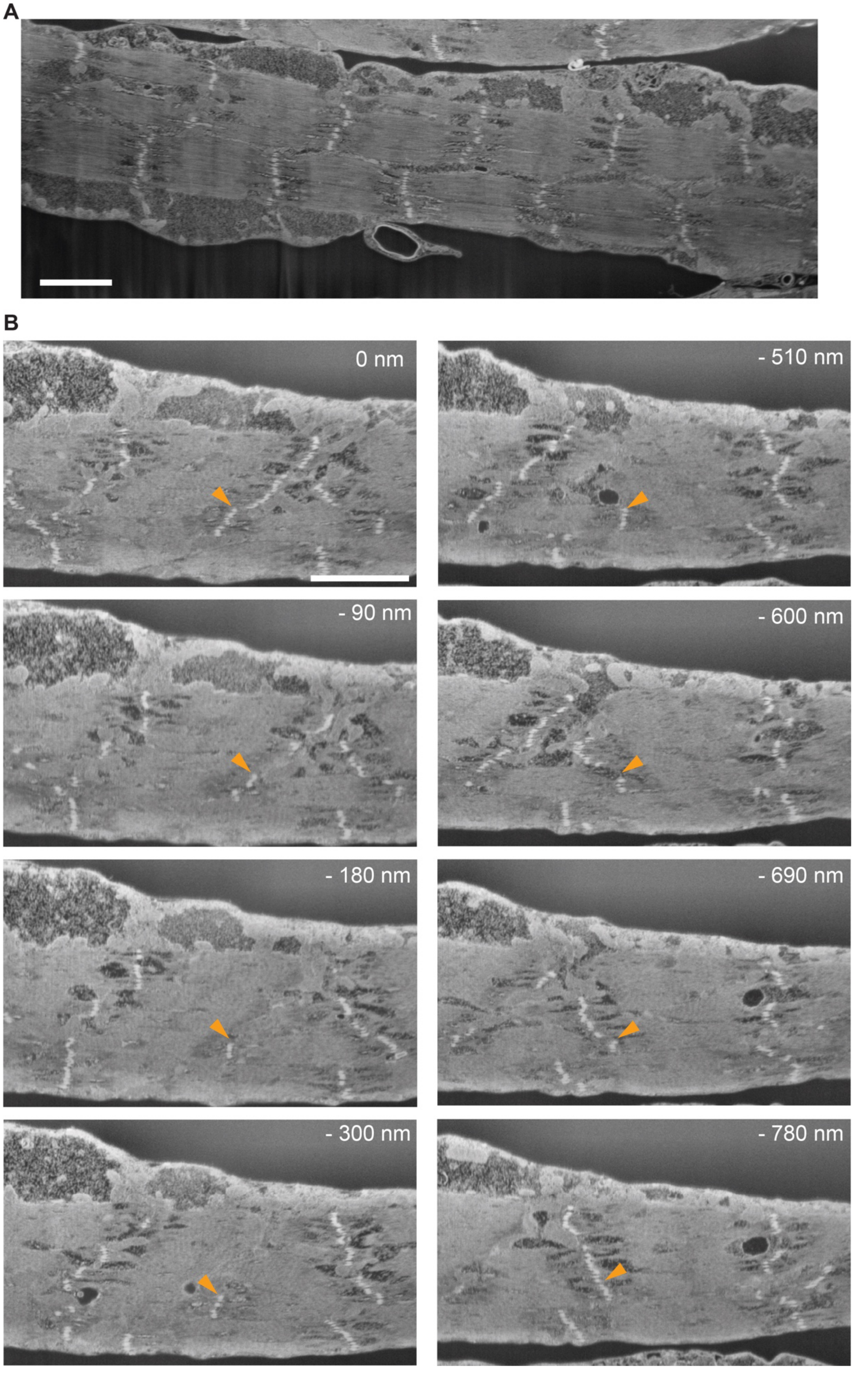
A
B
0 nm
- 90 nm
- 180 nm
- 300 nm
- 510 nm
- 600 nm
- 690 nm
- 780 nm

Fig. S8 – Examples of SEM slices of larval muscles.
(A) Representative SEM image of a *Drosophila* larval muscle with stacked myofibrils showing organized sarcomeres. (**B**) SEM images from various slices acquired from the same volume, identifying where an isolated Z-disc marked by an orange arrowhead is connected to the Z-disc from an adjacent sarcomere. The relative position of each slice in the volume is indicated relative to the top slice. Scale bars are 5 µm.

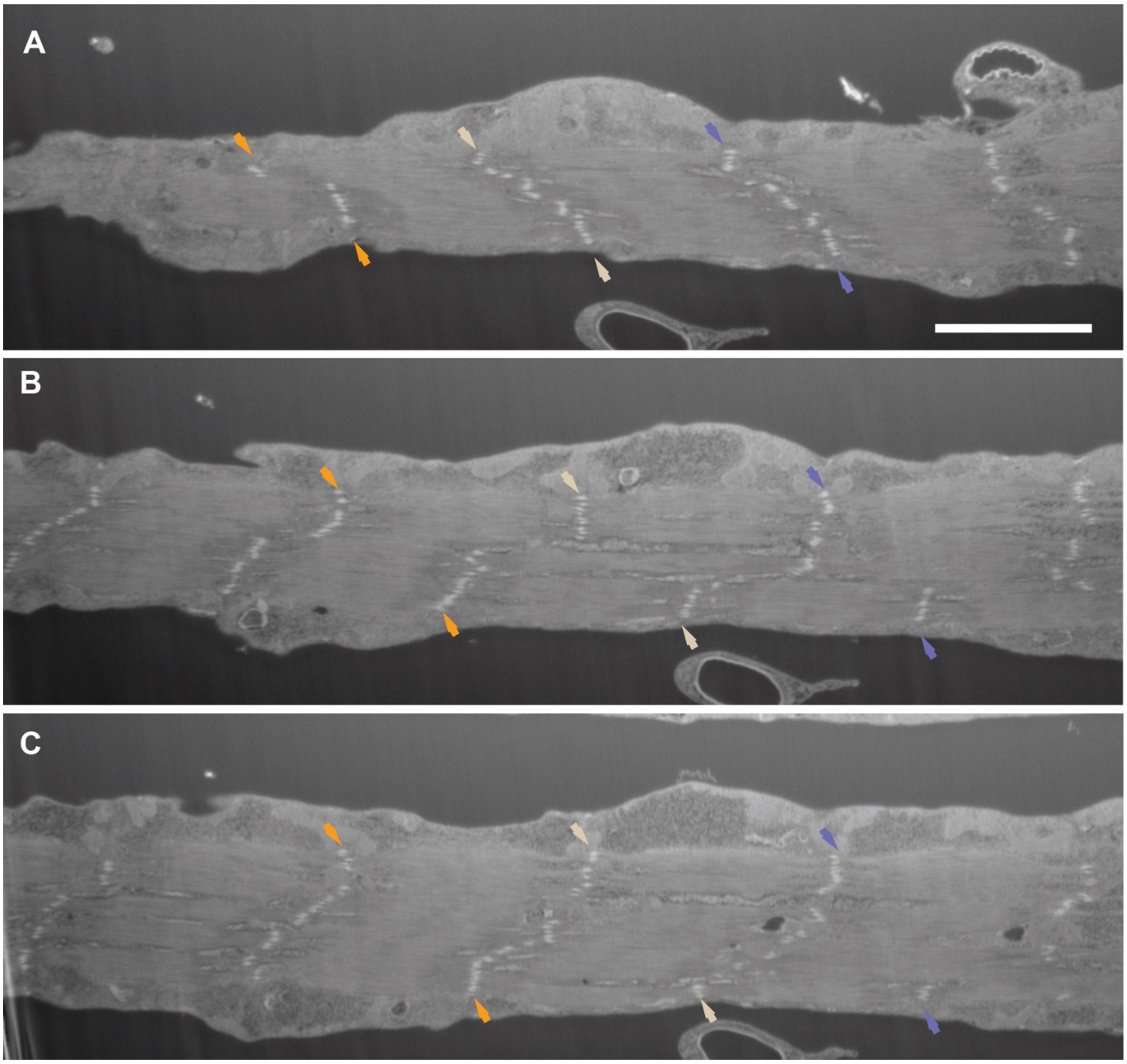

Fig. S9 – SEM reconstruction of larval muscles.
(A) SEM image at the surface of a larval muscle. Colored arrowheads indicate ends of Z-discs that are in the same register at the muscle edge. (**B**) SEM image from 300 nm deeper in the muscle, at which Z-discs have branched in a different direction. (**C**) SEM image from a further 240 nm deeper in the muscle, at which Z-disc connections are reformed between adjacent sarcomeres. This volume was reconstructed in Fig. 6A and Movie S9. Scale bar is 7 µm.

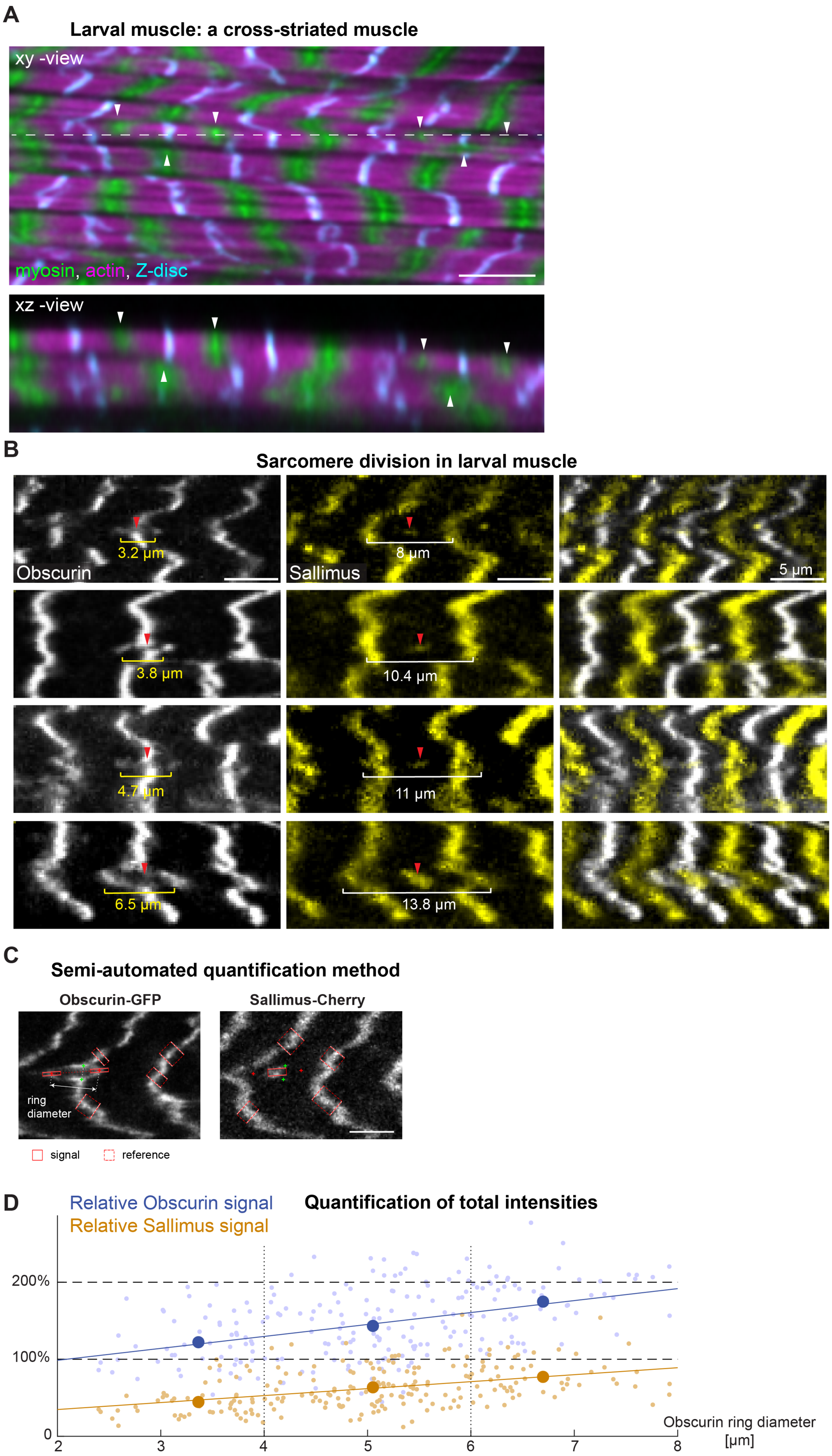
A
Larval muscle: a cross-striated muscle
xy -view
myosin, actin, Z-disc
xz -view
B
Sarcomere division in larval muscle
3.2 µm
8 µm
Obscurin
Sallimus
5 µm
3.8 µm
10.4 µm
4.7 µm
11 µm
6.5 µm
13.8 µm
C
Semi-automated quantification method
Obscurin-GFP
Sallimus-Cherry
ring diameter
signal
reference
D
Relative Obscurin signal
Relative Sallimus signal
Quantification of total intensities
200%
100%
0
2
3
4
5
6
7
8
Obscurin ring diameter [µm]

Fig. S10 – Sarcomeres in living larval muscles.
(**A**) High-resolution image of a fixed larval muscle expressing Mhc-GFP (green) stained for actin (phalloidin, magenta) and Sls (Sls-Nano2 cyan). xy-plane shown at the top and reconstructed xz-view at the bottom. Note that myosin stacks (green, marked by white arrowheads) can be separated in xy but remain connected in z. See also Movie S10. (**B**) Living larvae expressing Obscurin-GFP (white) and Sallimus-mCherry (yellow) in muscles. From top to bottom, dividing sarcomeres of increasing ring sizes were sorted. Note that the cross-striated myosin filament stacks segregate, while new Z-disc material (Sls) continues to be incorporated at increasing sarcomere length. **(C)** Illustration of the semi-automated quantification method used for (D). Shown is a single slice of a two-channel z-stack with Obscurin-GFP (left) and Sallimus-mCherry (right); green crosses: manually determined junction points joining the Obscurin ring to M-bands above or below; red crosses: marking the outermost points of the ring; solids red rectangles: regions for line scans of Obscurin and Sls signal; dashed red rectangles: regions for reference line scans used for normalization. (**D**) Intensity quantifications of dividing sarcomeres sorted by Obscurin-GFP ring width. Note that the relative intensity of Obscurin doubles during the division of one A-band to two A-bands and the amount of Sls increases to reach the amount of a regular sarcomere (gold: Sls, blue: Obscurin; small symbols: individual 'rings', large symbols: binned data with bin boundaries indicated by dashed lines; solid lines: linear regression; F-test versus constant model: Obscurin: p=1.3 10^{-11}, Sls: p=8.0 10^{-12}). All scale bars are 5 μm.

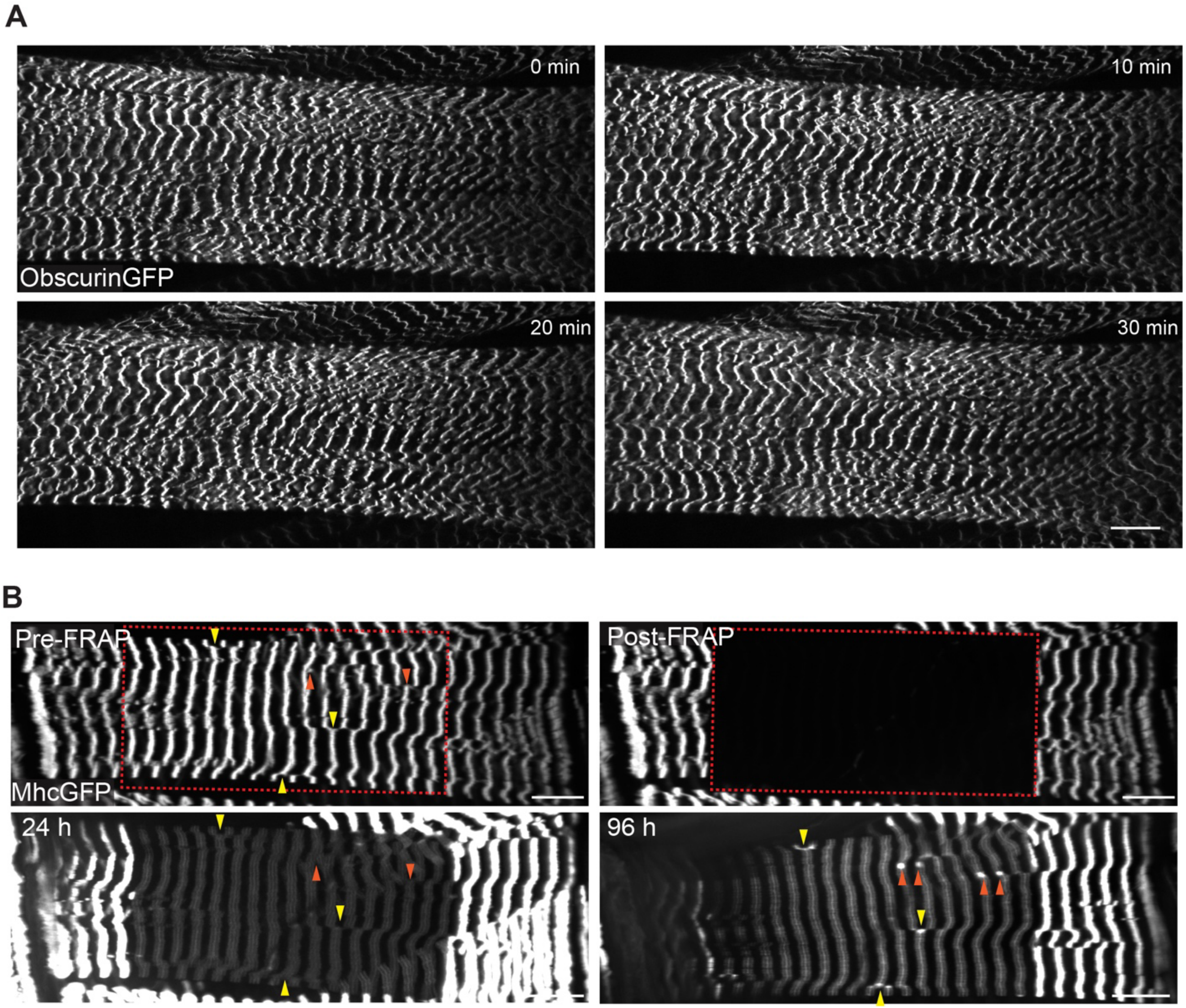

Fig. S11 – Live imaging of larval muscle and FRAP.
(A) Stills from a 30-min movie of a living anaesthetized L3 larva expressing Obscurin-GFP to label M-bands. Note that M-bands show little dynamics and sarcomere number remains constant (see Movie S11). (**B**) Mhc-GFP in living L3 larval muscle (dorsal oblique 2, DO2), before FRAP (left, red square) and after FRAP (right), as well as after 24 h (left, bottom) and 96 h (right bottom) of recovery in food. Note the incorporation of new Mhc-GFP in pairs marked by orange arrowheads or at myosin 'zippers' (yellow arrowheads). Scale bars are 20 µm.

Movie S1 (separate file). Live imaging of Sls-GFP at flight muscle ends.
Live imaging of 34 h APF pupa expressing Sls-GFP with a focus on the anterior flight muscle end, imaged every 3 minutes. Arrowheads follow the first 3 sarcomeres over time.

Movie S2 (separate file). Live imaging of Mhc-GFP at flight muscle ends.
Live imaging of 36 h APF pupa expressing Mhc-GFP with a focus on the anterior flight muscle end, imaged every 3 minutes. Arrowheads follow the first 3 sarcomeres over time.

Movie S3 (separate file). Three-dimensional visualization of traced myofibrils at 40 h APF. Animation of traced myofibrils identified in a *z*-stack of 40 h APF flight muscles stained with Sallimus in green (Sls-Nano2), myosin in blue (Mhc-GFP) and actin in red (phalloidin). Grey lines indicate the detected sarcomeres between 2 green dots.

Movie S4 (separate file). Three-dimensional visualization of traced myofibrils at 48 h APF. Similar to Movie S3 using a 48 h APF flight muscle sample.

Movie S5 (separate file). Animation of sarcomere division.
Animation of a dividing sarcomere using the model shown in Fig. 2E. Note that the daughter 1 sarcomere (Z1, M1, S1) segregates from daughter 2 sarcomere (S2, M2, Z2). At the end, S1 and S2 fuse and establish the new Z-disc.

Movie S6 (separate file). Live imaging of Mhc-GFP turnover in flight muscles.
Live imaging of Mhc-GFP expressing flight muscles at 36 h APF with spinning disc microscopy, imaged every 5 min. The white rectangle area was bleached and Mhc-GFP recovery was followed.

Movie S7 (separate file). Imaging and tracking of A-band division live.
Two-photon microscopy live imaging of Mhc-GFP expressing flight muscles at 36 h APF. Myosin stacks were automatically tracked along one myofibril marked with black crosses. The one marked with a red cross divides into two myosin stacks, starting at 0 minutes. These data were used to quantify distances in Fig. 5.

Movie S8 (separate file). Imaging of A-band divisions live.
Two-photon microscopy live imaging of two different pupae expressing flight muscles at 36 h APF. Dividing myosin stacks and their immediate neighbours were manually marked with arrowheads and followed over time. Note myosin filament stack divisions occur anywhere in the muscle cell.

Movie S9 (separate file). Three-dimensional reconstruction of larval muscle with SEM.
Scanning electron microscopy segmentation of larval Z-discs in a 3D volume starting from the muscle surface using the slices shown in Fig. S10.

Movie S10 (separate file). Three-dimensional view of larval muscle.
Z-stack of high-resolution images of a fixed larval muscle expressing Mhc-GFP (green) stained for actin (phalloidin, magenta) and Sls (Sls-Nano2 cyan). Note that M-bands and Z-stacks are often segregated in 2D, but stay largely connected in 3D.

Movie S11 (separate file). Live imaging of anaesthetized larvae.
Live imaging of an Obscurin-GFP expressing dorsal oblique 2 muscle (DO2) in an anaesthetized larva, imaged every 2 minutes with a spinning disc confocal microscope. Note that M-bands are stable over the entire movie.

Movie S12 (separate file). Dynamic recovery of Mhc-GFP after FRAP.

Z-stack of living larva that expresses Mhc-GFP in green and Sls-Cherry in magenta, 7 hours after FRAP recovery with feeding. Note that Mhc-GFP does recover in pairs marked by arrowheads in the individual planes.

Data S1 (separate file) – Data related for Fig. 1

Data S2 (separate file) – Data related for Fig. 3

Data S3 (separate file) – Data related for Fig. 4 and Figs. S3, S4, S6

Data S4 (separate file) – Data related for Fig. 5 and Fig. S7

Data S5 (separate file) – Data related for Fig. 6 and Fig. S10
